# Minimal chemical modification enables alpha/beta radiolabeling of sacituzumab govitecan for targeted therapy in high grade serous ovarian cancer

**DOI:** 10.1007/s00259-026-07820-7

**Published:** 2026-02-28

**Authors:** Angelique Loor, Kyeara N. Mack, David Bauer, Aidan Ingham, Edwin C. Pratt, Jason S. Lewis

**Affiliations:** 1 Molecular Pharmacology Program, Memorial Sloan Kettering Cancer Center, New York, NY USA; 2 Department of Pharmacology, Weill Cornell Graduate School of Medical Sciences, Weill Cornell Medicine, New York, NY USA; 3 Department of Radiology, Memorial Sloan Kettering Cancer Center, New York, NY USA; 4 Stony Brook University Cancer Center, Stony Brook, NY USA

**Keywords:** TROP-2; PET imaging, Radioimmunotherapy, Ovarian cancer, Targeted alpha therapy

## Abstract

**Purpose:**

Ovarian cancer (OC) is frequently diagnosed at late stages after metastasis and chemorefractive leading to poor survival outcomes. There is a critical need for earlier detection and novel antigen-targeted therapies to improve patient survival at all stages. TROP-2 is a transmembrane glycoprotein overexpressed in many cancers and presents a promising target for OC. Recently, antibody-drug conjugates such as sacituzumab govitecan (SG) and datopotamab deruxtecan have been approved for various TROP-2-positive malignancies such as metastatic breast, lung, or urothelial cancer. However, diagnosis is based on prior therapy failure and TROP-2 therapy focused on antibody-drug conjugates.

**Methods:**

Minimal radiotheranostic versions of SG were developed for immunoPET imaging with [^89^Zr]Zr-DFO-SG in flank and intraperitoneal OVCAR3 implants. IHC was also done to identify other OC models that express Trop2. Radiotherapy variants of SG [^177^Lu]Lu-DTPA-SG, [^225^Ac]Ac-mcp-Direct-SG, or [^225^Ac]Ac-mcp-Click-SG were also made and tested for efficacy alongside the standard of care SG.

**Results:**

[^177^Lu]Lu-DTPA-SG delayed tumor recurrence for up 6–8 weeks post-treatment and retreatment prolonged overall survival to 21 weeks matching standard of care SG dosing. Between two linkers, [^225^Ac]Ac-mcp-Direct-SG was found to yield a superior minimal conjugation of SG than [^225^Ac]Ac-mcp-Click-SG, with better tumor targeting by biodistribution and prolonged reduction in OC over 30 weeks.

**Conclusion:**

Ultimately, utility of SG was improved through minimal modification of the ADC and applied a “treat what you see” approach to TROP-2-positive OC. By using a new tetrafluorophenyl linkage of macropa, [^225^Ac]Ac-mcp-Direct-SG greatly reduced tumor burden in OC with most mice surviving and tolerating the therapy.

**Supplementary Information:**

The online version contains supplementary material available at 10.1007/s00259-026-07820-7.

## Introduction

Ovarian cancer (OC) is characterized by its aggressive, heterogeneous tumor microenvironment and its tendency to present at advanced stages [1, 2] and OC patients have the highest mortality rate of all gynecologic cancers [3]. Approximately 90% of OC’s are epithelial, with most high-grade serous tumors being linked to the worst prognosis. Within OC there are options beyond surgery and chemotherapy that include targeted small molecule therapies like PARP, NTRK, and BRAF inhibitors. Antibody based therapies are also used targeting VEGF (bevacizumab), HER2 (trastuzumab) as well as success with newer antibody drug conjugates (ADCs) including trastuzumab-deruxtecan [4] and mirvetuximab soravtansine (MIRV) targeting folate receptor alpha-positive, platinum-resistant disease [5]. The Phase III results from the MIRASOL trial demonstrated that MIRV improved both overall survival and progression-free survival compared to chemotherapy alone [6]. However, there is still an unmet need for ADCs targeting different antigens and or utilizing alternative drug payloads to address this complex disease more comprehensively.

One target of interest for OC is the human trophoblast cell surface antigen-2 (TROP-2) [7]. TROP-2 is a 46 kDa membrane-bound glycoprotein expressed in various epithelial cancers, including OC, triple-negative breast cancer, and pancreatic cancer [8]. TROP-2 has been shown to play a multifaceted role in OC development through the promotion of proliferation, metastasis, and angiogenesis [8, 9]. Among other reports, administering the ADC, sacituzumab govitecan (SG) to TROP-2-positive ovarian carcinosarcomas, reduced tumor burden and improved overall survival [10]. SG contains the irinotecan metabolite SN-38 via a maleimide-polyethylene-glycol-acid pH-sensitive carbonate linker [11]. With approximately 7.6 SN-38 molecules per TROP-2 antibody, there is still potential for bioconjugation and has been explored recently for imaging other cancers [12, 13]; however, the degree of labeling can dramatically influence the distribution of the antibody [14], particularly an already laden ADC. The success of SG in the clinic could benefit from a noninvasive approach to predict response and patient stratification but do so while minimally adding to the degree of conjugation. While ADCs hold promise for treatment, they frequently encounter obstacles stemming from tumor heterogeneity, drug release, and drug resistance [15] and new therapeutic strategies are needed.

**Fig. 1:**
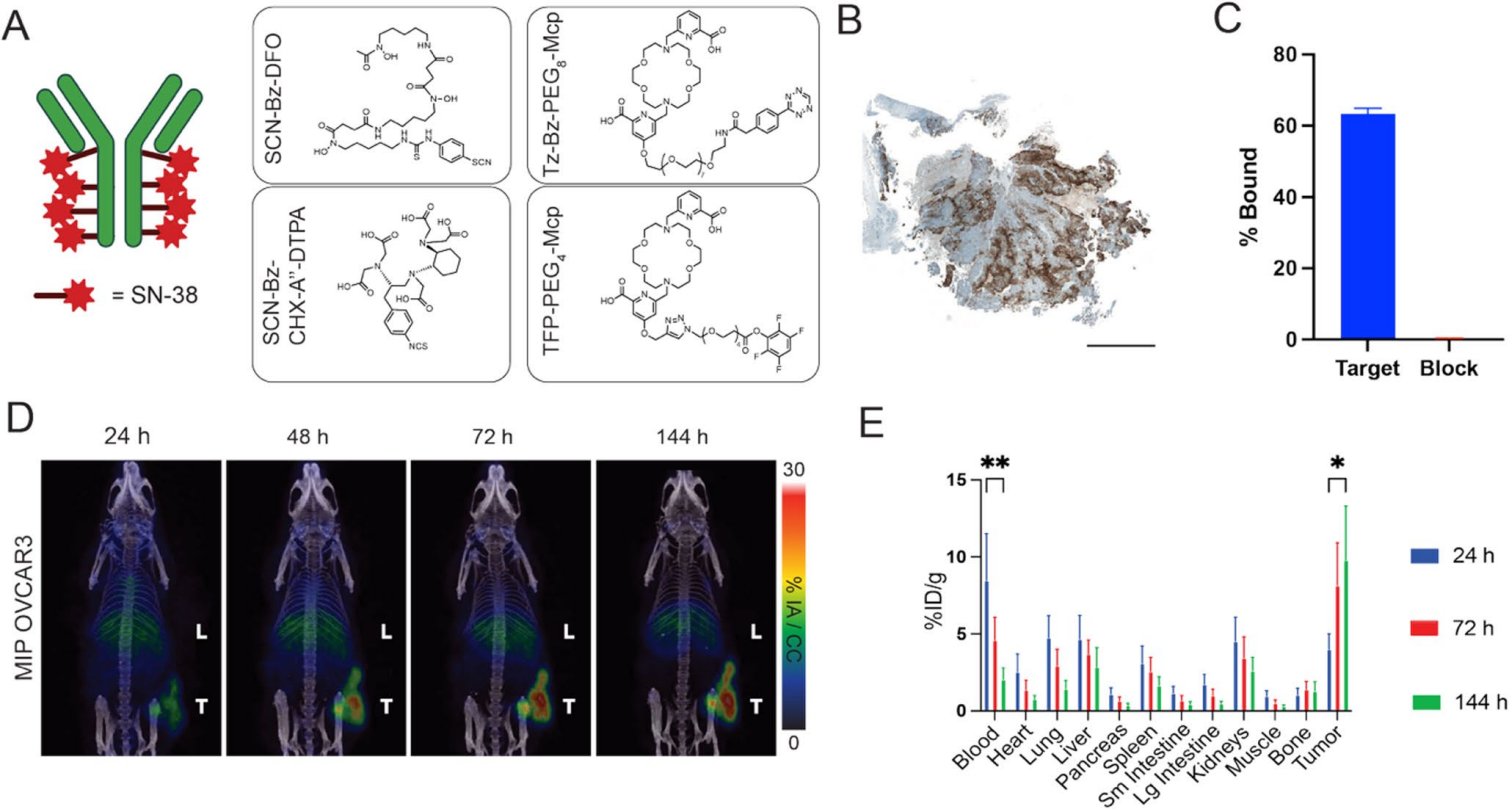
OVCAR3 cell line is TROP-2-positive and [^89^Zr]Zr-DFO-SG is readily taken up in vivo. **A**) SG contains up to 8 SN-38 warheads yet can still be paired with different chelators for imaging and radioimmunotherapy, utilizing SCN-Bz-DFO for PET imaging, SCN-Bz-CHX-A’’-DTPA, Tz-Bz-PEG_8_-Mcp, or TFP-PEG_4_-Mcp for therapy. **B**) Immunohistochemistry of OVCAR3 tumors reveals high expression of TROP-2. **C**) [^89^Zr]Zr-DFO-SG was taken up in OVCAR3 cells and blocked by an excess of 100-fold of unlabeled SG. **D**) PET/CT images at 24-, 48-, 72-, and 144- h post administration of [^89^Zr]Zr-DFO-SG accumulated mainly in OVCAR3 tumors (T) and non-specific liver (L). E) Ex vivo biodistribution of 144 h shows high blood activity at 24 h decreased over time indicating clearance (**p < 0.01). Tumor uptake increased significantly by 144 h (*p < 0.05), confirming specific accumulation and retention, suggesting favorable pharmacokinetics and stability for imaging applications. B) Scale bar = 2 mm. C, E) Bar = mean with error = SEM, *n* = 3 replicates

Previously, high-grade serous OCs were identified by hypoglycosylated MUC16 expression using an immunoPET conjugate with the AR9.6 antibody [16]. Theranostic conversion to a lutetium-177 (^177^Lu) or actinium-225 (^225^Ac) based radiotherapy provided significant responses in preclinical models [16, 17]. Here the use of an antibody as an imaging and therapy agent, or theranostic, to treat what is seen presents an effective strategy to identify and control recurrent tumors and further survival outcomes. Furthermore, the use of directed beta or alpha radiation allows targeting of cancers cells without the need for a drug release or internalization [18]. Integrating ADCs with radiotherapy presents an opportunity to surmount each of these challenges by providing an extended therapeutic range over ADCs and including the adjuvant release of the therapeutic warhead. Previous approaches have involved separate administrations of the ADC and non-targeted radiation [19]. Knowing that the synergistic potential of radiation and ADC therapy has been evident in numerous preclinical models, the goal was to convert SG to an ADC theranostic using a minimal conjugation strategy to add as few chelates per ADC. Here minimal DFO, DOTA, and macropa conjugates were created to create an ADC theranostic (Fig. 1a) for OC. Minimal modification provides molecular imaging and radiotherapy alongside drug release with the ADC and by not overloading the antibody, liver uptake is minimalized.

## Materials and methods

The Supplementary Information covers cell-line authentication and culture conditions, serum-stability, intraperitoneal implantation, immunohistochemistry, cell and bead-binding assays, autoradiography, pathology analysis, and the synthesis and characterization of mcp-PEG_4_-TFP.

### Radiochemistry

#### Zirconium-89 bioconjugation and radiolabeling for PET

SG (Gilead) was reconstituted (10 mg/mL, sterile saline), and conjugated to p-NCS-Bn-Deferoxamine (DFO) (Macrocyclics, B-705); in chelex-treated PBS (pH 8.7) using a 6-fold molar excess of deferoxamine (10 mg/mL in DMSO), incubated at 37 °C for 1 h, purified via a PD-10 column, and concentrated via a 30 kDa Amicon filter. Radiolabeling of SG-DFO with [^89^Zr]Zr-oxalate (1 M oxalic acid) was achieved. By adjusting the pH of solution to ~ 7.4 with HEPES buffer (1 M), followed by addition of antibody, and incubation in thermomixer at 37 °C for 1 h. Radiochemical conversion was assessed via radio-iTLC with EDTA buffer (50 mM, pH 5.5). Serum stability was assessed over 7 days at 37 °C in human serum (100167-WEB).

#### Lutetium-177 and Actinium- 225 bioconjugations and radiolabeling for radiotherapies

SG was buffer exchanged with chelex-treated PBS (pH 8.7) and conjugated to p-NCS-CHX-A”-DTPA (DTPA) (Macrocyclics, B-355), which was in a 6-fold excess, to yield DTPA-SG. Lutetium-177 radiolabeling was performed in 250 mM ammonium acetate (pH 5.5), incubated at 37°C for 1 h, and analyzed via radio-iTLC. Doses of 9.25 MBq (20 µg) and 18.5 MBq (40 µg) were prepared of [^177^Lu]Lu-DTPA-SG.

For Actinium-225 studies, SG was conjugated to macropa (mcp) via two-strategies: (i) click chemistry using a trans-cyclooctene (TCO)-modified SG and mcp-PEG_7_-Tz (17), or (ii) direct conjugation with tetrafluorophenyl ester (mcp-PEG_4_-TFP). Conjugations to SG were performed in chelex-treated PBS (pH 8.9) for 4 h at a 6-fold molar excess of mcp-PEG_4_-TFP, or a 30-molar excess of NHS-TCO (axial). Products were purified by PD-10 and Amicon 30 kDa concentrated. Actinium-225 radiolabeling was performed at 37 °C: mcp-Direct-SG in HEPES (37 kBq, 20 µg and 25.9 kBq, 14 µg), and mcp-Click-SG in 0.25 M ammonium acetate (pH 5.5) for 15 min, then neutralized and clicked to TCO-SG (37 kBq, 20 µg). Radiochemical conversion via radio-iTLC (0.4 M citrate buffer, pH 4) at 1 and 24 h post-iTLC with greater than 99% actinium-225 at the origin.

### Animal studies

#### Xenograft OVCAR3 model

All IACUC-approved procedures (MSKCC #08-07-013) used female nude mice (6–8 weeks, Charles River) implanted on the right flank with 10 million OVCAR3 cells in 150 µL 1:1 medium/BD Matrigel. Initial xenografts were given an additional 5 million OVCAR3 cells implanted as published previously [16]. Tumors reaching 100–350 mm^3^ were assigned to imaging (*n* = 5) or therapy (*n* = 10) cohorts. At start of therapy, cohort averages were between 100 and 350 mm^3^, though some individual tumors were larger than 350 mm with only one in the entire study over 550 mm^3^.

#### PET and Cerenkov imaging

Female nude mice (RA00077606) bearing subcutaneous tumors received 9.2 MBq, 20 µg of [^89^Zr]Zr-DFO-SG, with imaging at 24–144 h post-injection on a Siemens Inveon PET/CT capturing 40 million events per mouse per timepoint. Reconstructed images (2DOSEM 512 binning) were then calibrated to percent injected activity per cubic centimeter. For the Lutetium-177 study, mice were imaged 24 h after administration of [^89^Zr]Zr-DFO-SG to confirm positivity. After second dose of [^177^Lu]Lu-DTPA-SG, mice were imaged 24 h post-injection via Cerenkov luminescence (IVIS, open filter, 300 s). Tumor radiance was reported in p/s/cm^2^/sr using Living Image V 4.2.

#### Radioimmunotherapy (RIT)

Mice bearing OVCAR3 tumors (100–350 mm^3^) were enrolled into five therapy studies (*n* = 10):

- Control – one dose of 20 µg of SG.
- Standard of Care – 180 µg of SG on days 1, 7, and 14; cycle repeated monthly after 1-week break.
- SG ^177^Lu Therapy – 9.25 MBq (20 µg) followed by 18.5 MBq (40 µg) of [^177^Lu]Lu-DTPA-SG.
- Click-SG ^225^Ac Therapy – 37 kBq, 20 µg of [^225^Ac]Ac-mcp-Click-SG.
- Direct-SG ^225^Ac Therapy – 37 kBq (20 µg) followed by 25.9 kBq (10 µg) of [^225^Ac]Ac-mcp-Direct-SG.

Complete blood count (CBC) parameters (white blood cells (WBC), red blood cells (RBC), and platelets) were monitored weekly in *n* = 3 mice/group. Tumor sizes and body weight were recorded twice weekly. Mice whose weight decreased received supplemental DietGel. Therapy endpoints were 2,000 mm^3^, petechia, and > 20% body weight loss. Tumor dimensions were measured with vernier caliper, and TV calculated by:

$$\:TV=\left(\frac{4\mathrm\pi}3\right)\left(\frac\alpha2\right)^2\left(\frac b2\right)$$

TV = tumor volume (mm)^3^

α = longest axis of the tumor (mm)

*b* = axis perpendicular to the longest axis (mm), α (mm)

#### Ex vivo biodistribution

Ex vivo biodistribution analyses were performed on separate cohorts of xenografted mice that were administered [^89^Zr]Zr-DFO-SG, [^225^Ac]Ac-mcp-Direct-SG, or [^225^Ac]Ac-mcp-Click-SG. For serial biodistribution studies with [^89^Zr]Zr-DFO-SG, mice were injected with 9.25MBq (20 µg). Mice (*n* = 3) were euthanized at 4, 24, and 144 post-injection. For the [^225^Ac]Ac-mcp-Direct-SG and [^225^Ac]Ac-mcp-Click-SG cohort, mice were injected with 37 kBq (20 µg). Mice (*n* = 3) were euthanized at 4, 24, 72 and 168 post-injection. Organs harvested include blood, heart, lung, liver, spleen, pancreas, small intestine, large intestine, kidneys, muscle, bone, and tumor. Organs were weighted, and activity was measured using a Perkin Elmer Wizard 3 series gamma counter with the respective radionuclide protocol. Values were reported as % injected activity per gram of tissue.

### Statistical analysis

Data were analyzed using GraphPad Prism v10. Unpaired, two-tailed *t* tests assessed bead and cell-binding; one-way ANOVA with Tukey-Kramer correction was used for organ uptake in biodistribution experiments. Survival was analyzed via a log rank (Mantel–Cox) test. A *P* value less than 0.05 was considered statistically significant.

## Results

### Effective targeting of TROP-2-positive OC using [^89^Zr]Zr-DFO-SG

Based on prior work treating OC with SG [10], Trop-2 positivity was examined in several OC cell line xenograft tumors by IHC, identifying OVCAR3 with the strongest though varied staining (Fig. 1B, Supplemental Fig. 1). SG was thus conjugated with SCN-Bz-DFO for Zirconium-89 yielding [^89^Zr]Zr-DFO-SG with > 98% radiochemical conversion and stability was observed in human serum to be greater than 96% at 7 days (Supplemental Fig. 2A, B). SG was found by MALDI to lose about 2.6 SN-38 molecules when stored at 4 C for over a month (Supplemental Fig. 2C-D), thus conjugates were prepared fresh for each study. Maintaining a minimal footprint, DFO-SG was determined to have ~ 0.5 DFO chelates per SG (Supplemental Fig. 1E). To confirm [^89^Zr]Zr-DFO-SG immunoreactivity, a cell uptake assay with OVCAR3 cells show binding (63%) with nominal uptake in the SG block (0.2%) (Fig. 1C). Moving in-vivo [^89^Zr]Zr-DFO-SG in nude mice bearing OVCAR3 tumors, PET/CT imaging showed localized delivery to flank tumors observed 24 h post-injection with some liver uptake, with maximal tumor uptake observed at 144 h (Fig. 1D). These findings were corroborated by terminal biodistribution, with tumor uptake increasing to 144 h post with clearance from all other tissues measured (Fig. 1E). In a parallel model [^89^Zr]Zr-DFO-SG was administered once a month between one and five months post implant in nude mice with OVCAR3 IP tumors. PET/CT was used to assess IP tumor growth and confirm target avidity with time. Monthly administrations of [^89^Zr]Zr-DFO-SG identified avid lesions through month 5 (Supplemental Fig. 3A) and administration alone of 20 µg SG monthly was not therapeutic. Ex vivo autoradiography confirmed tumor uptake was more avid than liver with some heterogeneity within the tumor (Supplemental Fig. 3B), consistent with IHC findings in Fig. 1B.

### [^177^Lu]Lu-DTPA-SG demonstrates tumor suppression, and overall survival

**Fig. 2:**
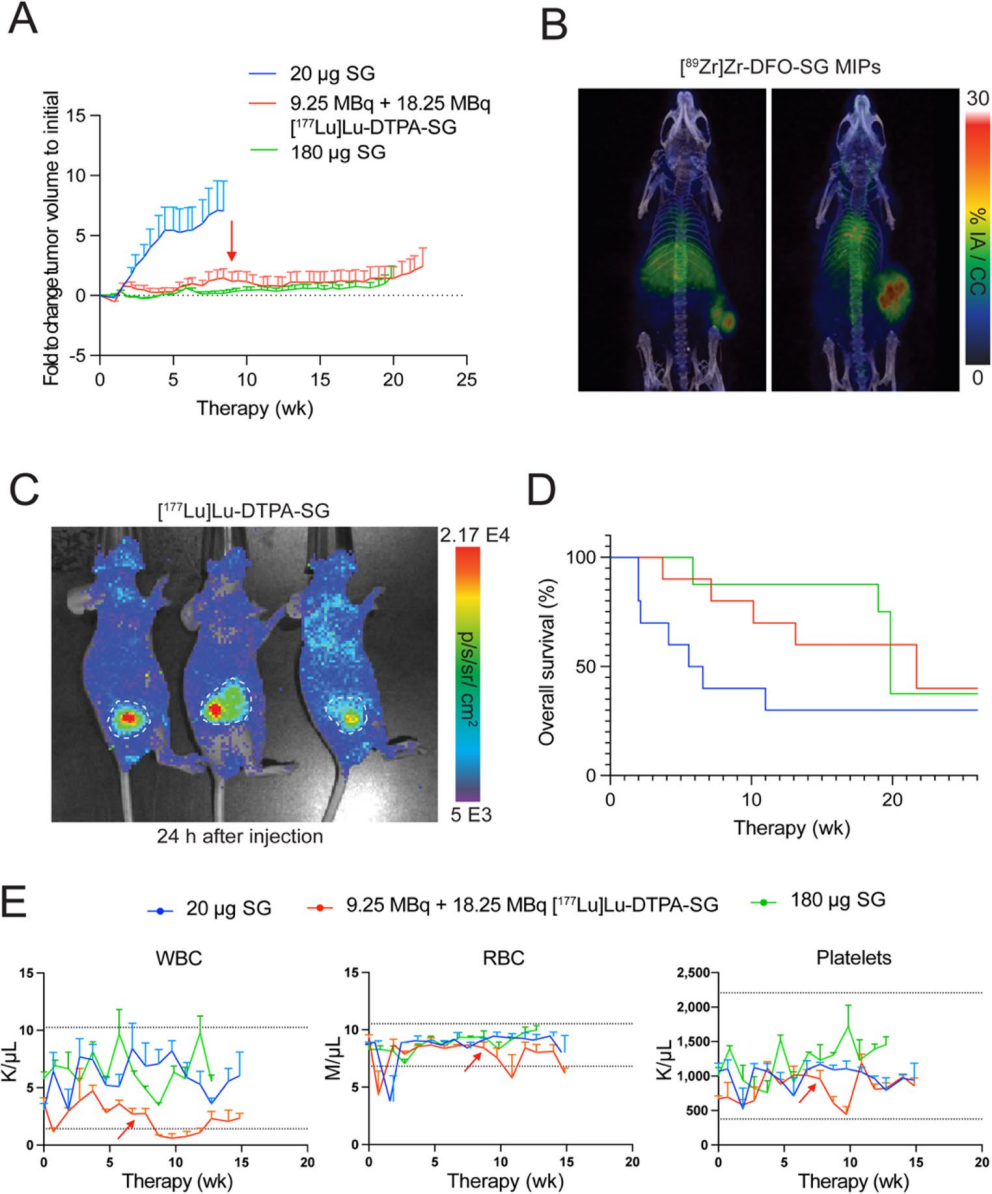
Treatment with [^177^Lu]Lu-DTPA-SG reduced tumor burden, increased survival for mice. (**A**) Fold change in growth from the start of therapy shows two doses of 20 µg [^177^Lu]Lu-DTPA-SG were similar to the standard of care (180 µg weekly) SG. (**B**) Tumors in the [^177^Lu]Lu-DTPA-SG cohort began to regrow, prompting a reassessment of TROP-2 expression, which confirmed tumors remained TROP-2-positive as both small (left) and larger tumor sizes (right). (**C**) Administration of a second higher dose of [^177^Lu]Lu-DTPA-SG was confirmed by Cerenkov luminescence imaging 24 h post-injection. (**D**) The median survival of [^177^Lu]Lu-DTPA-SG was 21 weeks compared with 6 weeks for the 20 µg SG dose and similar to the standard of care weekly 180 µg SG cohort at 20 weeks. (**E**) CBC showed minor decreases in all parameters following administration of [^177^Lu]Lu-DTPA-SG which recovered 3–4 weeks after. Data is presented as mean ± SEM, with n = 3 mice per group for CBC analysis. Arrow indicates the administration of the second dose

[^177^Lu]Lu-DTPA-SG was synthesized with radiochemical conversion > 98% (Supplemental Fig. 2A). A bead binding assay with OVCAR3 confirmed preserved antigen specificity to TROP-2 (Supplemental Fig. 4). Survival therapy was initiated in OVCAR3 tumor-bearing nude mice, with mice being injected either 9.25 MBq / 20 µg [^177^Lu]Lu-DTPA-SG, 20 µg of SG (control), or 180 µg of SG weekly as standard of care. Mice treated with 20 µg of SG showed minimal impact on tumor suppression due to inadequacy of ADC dosing as seen before in the IP model. Meanwhile, weekly administration of 180 µg SG, slowed tumor progression compared, but tumors continued to advance over time. Mice in the 9.25 MBq [^177^Lu]Lu-DTPA-SG showed static tumor size, similar to the 180 µg standard of care but tumors regrew 6–8 weeks post-treatment (Fig. 2A).

To confirm eligibility for a second round of radiotherapy, 9.25 MBq [^177^Lu]Lu-DTPA-SG mice were administered [^89^Zr]Zr-DFO-SG (8 weeks after initial treatment). PET/CT imaging 24 h post-injection confirmed TROP-2 expression in tumors, encouraging retreatment with [^177^Lu]Lu-DTPA-SG (Fig. 2B). Mice were administered a higher dose of 18.5 MBq [^177^Lu]Lu-DTPA-SG given the lack of tumor regression with the prior 9.25 MBq dose, and Cerenkov luminescence confirmed tumor delivery (Fig. 2C). Two mice treated with the higher dose displayed no visible tumors, showing complete regression. The administration of the second and higher [^177^Lu]Lu-DTPA-SG dose extended overall survival to 21 weeks, compared to 6 weeks in the control SG once group (Fig. 2D). Individual plots of mouse tumor sizes show heterogeneous responses with some tumors regressing (Supplemental Fig. 5A), while others exhibited slow progression in the standard of care and rapid growth in the SG only cohorts (Supplemental Fig. 5C, E). Weights in all cohorts increased with age (Supplemental Fig. 5B, D,F). Overall, retreatment of [^177^Lu]Lu-DTPA-SG demonstrated delayed tumor growth and extended overall survival but similar to the standard of care administration with SG alone. Weekly blood analysis showed administration of [^177^Lu]Lu-DTPA-SG led to a transient dip in WBC, RBC, and platelet counts which declined in a dose-dependent manner but recovered to within normal range by 3–4 weeks post-injection. RBC and platelets remained within range, whereas WBC counts dropped after administration of the second dose (arrow) but recovered within 4–5 weeks (Fig. 2E).

At the study endpoint, seven mice were submitted for pathology and serum chemistry analysis. In all mice, serum chemistry revealed mild AST elevations, with one mouse exhibiting myelosuppression. Although platelets and WBC counts remained within normal ranges, pathology demonstrated splenic erythroid hyperplasia, which is consistent with reticulocytosis. Collectively, these results indicate that the observed hematological toxicity was dose-dependent and reversible, with no evidence of permanent organ damage (see supplemental information for additional details).

### [^225^Ac]Ac-mcp-Direct-SG cohort exhibits superior tumor suppression and increased survival

Given the inability with two doses of [^177^Lu]Lu-DTPA-SG to regress tumors, and the time required to produce a new cohort of OVCAR3 bearing mice, alpha therapy was next considered. Actinium-255 (^225^Ac) was used based on the decay chain and daughter release [20] yielding several therapeutic hits, for an improved efficacy and therapeutic outcome [21]. Previous ^225^Ac studies have used click chemistry to overcome synthesis or distribution limitations [22] particularly related to ^225^Ac labeling conditions necessary with DOTA. While newer chelators such as macropa(mcp) [23] have helped improve labeling efficiency with ^225^Ac over DOTA, the difference in linker via direct tetrafluorophenyl (TFP) ester or TCO-Tz click chemistry was unknown. Previous work established a 30-fold TCO conjugation would yield several handles on a TROP-2 antibody for Tz click, and with SG this method yielded 8.3 TCOs on average (Supplementary Fig. 2F). By targeting only 40% of the available TCOs with mcp-PEG_7_-Tz, on average 3 chelates would be added per TCO-SG with no free mcp-PEG_7_-Tz expected. Synthesis of [^225^Ac]Ac-mcp-direct-was achieved through conjugation of mcp to SG via the mcp-PEG_4_-TFP ester (Supplemental Methods and Scheme). Radiochemical conversion for both mcp variants was greater than 99% (Supplemental Fig. 2A) and MALDI confirmed that with TFP conjugation 1.6 chelates were found per SG (Supplemental Fig. 2G). A bead binding assay again confirmed that both [^225^Ac]Ac-mcp-SG constructs bind specifically to TROP-2 in vitro (Supplemental Fig. 2).

**Fig. 3:**
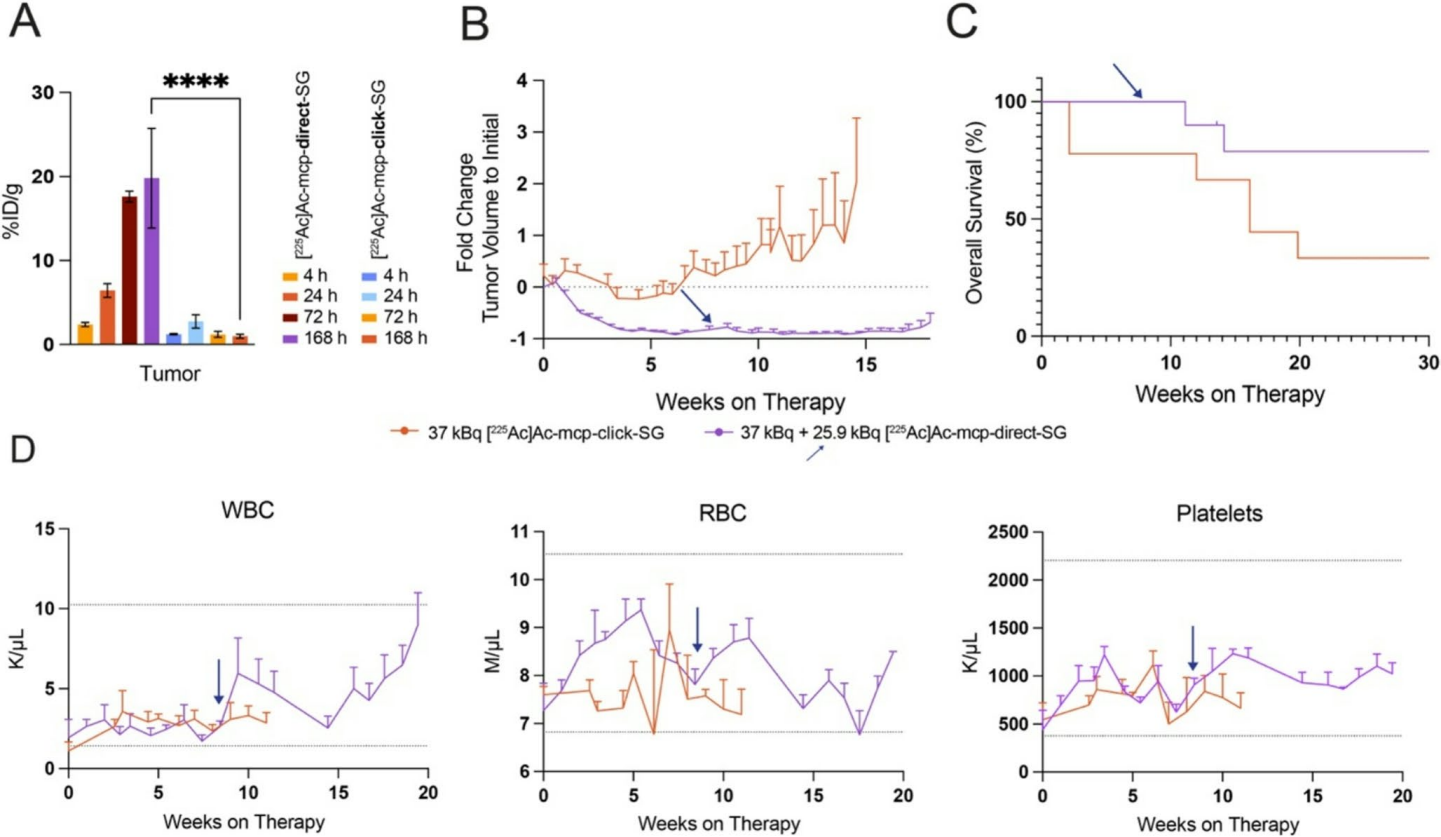
Greater tumor suppression, increased survival by change of macropa linker with SG. (**A**) Terminal biodistribution of key organs (Supplemental Fig. 6) and OVCAR3 xenografts at 4, 24, 72, and 168 h post-injection showed prolonged tumor retention for the direct cohort in contrast to the click cohort which displayed low/moderate targeting. (**B**) From the start of therapy, the directly conjugated SG (purple), even though retreated at week 9 (arrow), exhibited a substantial reduction in tumor burden compared to the clicked on macropa (orange), confirming biodistribution results in A). (**C**) Median survival was 16 weeks for the click cohort, while survival for the direct group was extended and undefined. (**D**) All main hematological parameters in the direct and click cohorts were within range (mean ± SEM, n = 3 mice per group per week). Blue arrow indicates administration of second dose. n = 5 mice per group for A while n = 10 mice were used for B-C. Bar=mean ± SEM. Arrow indicates administration of second dose for [^225^Ac]Ac-mcp-Direct-SG. **** p = <0.0001.

With the new mcp TFP and TCO-Tz linker chelates, ex-vivo biodistribution analysis was conducted at 4, 24, 72, and 168 h to compare the in vivo performance of the radioimmunoconjugates. The [^225^Ac]Ac-mcp-Direct-SG exhibited distribution across all tissues and showed mean tumor uptake of 19.80 ± 5.937%ID/g at 168 h, reflecting the localization of the radioimmunoconjugate to the tumor while minimal uptake was seen with [^225^Ac]Ac-mcp-Click-SG with the majority of the activity deposited in the liver and spleen as early as 4 h post injection (Fig. 3A, Supplemental Fig. 6). Extrapolating values from the biodistribution for therapy with a trapezoidal model shows a clear advantage for using [^225^Ac]Ac-mcp-Direct-SG (Supplemental Table 1), with the majority of the dose delivered to the tumor with the next organ receiving only 20%. However, for [^225^Ac]Ac-mcp-Click-SG organs such as the liver and spleen would receive the highest doses.

Both conjugates were moved into a therapy with OVCAR3 tumor-bearing nude mice. Mice in the [^225^Ac]Ac-mcp-Direct-SG cohort exhibited a pronounced reduction in tumor growth while [^225^Ac]Ac-mcp-Click-SG saw static tumor sizes for about 6 weeks before regrowth (Fig. 3B). Tumor reoccurrence was observed in *n* = 3 mice in the [^225^Ac]Ac-mcp-Direct-SG group, which were retreated and responded to therapy. The median survival for the [^225^Ac]Ac-mcp-Direct-SG cohort remained undefined, with most mice alive with minimal to no palpable tumors while [^225^Ac]Ac-mcp-Click-SG treated mice had a mean survival of 16 weeks (Fig. 3C). Blood analysis in both cohorts showed no deviations from the normal range (Fig. 3D).

At study end, surviving mice were submitted to pathology. Pathology identified flank carcinomas in all three [^225^Ac]Ac-mcp-Click-SG mice submitted whereas three mice in the [^225^Ac]Ac-mcp-Direct-SG group had no identifiable tumors. In addition, no evidence of metastasis was observed in any examined tissues. In the [^225^Ac]Ac-mcp-Click-SG group one mouse had marked elevation of BUN suggesting early-stage renal azotemia, and in the liver hepatocellular degeneration and hypertrophy was also observed all three mice. Neither of these findings were observed in mice receiving [^225^Ac]Ac-mcp-Direct-SG.

## Discussion

OC remains one of the deadliest gynecological malignancies, with a five-year survival rate below 50% due to late-stage diagnosis and therapeutic resistance to conventional treatment [24]. Despite the advancements in surgery and chemotherapies, effective treatments for recurrent and metastatic OC remain limited, emphasizing the need for new novel targets [25]. TROP-2, an aggressive pro-tumoral glycoprotein, is overexpressed in various cancers [8], including OC. Administration of SG does not require prior confirmation of TROP-2 expression in the cancers selected due to the assumption that all will be positive. However this remains to be seen in cancers outside of breast and lung; therefore, immunoPET strategies and companion theranostics can help identify patient eligibility for TROP-2 targeted therapy [26, 27] when histology status is unknown and likely heterogenous. In addition, as a surface antigen that internalizes, TROP-2 is an ideal theranostic target with the potential to improve diagnostic accuracy and treatment outcomes, particularly for OC patients with late diagnosis and high incidence of recurrence [28].

Here the FDA-approved ADC, SG, was repurposed into a theranostic agent capable of imaging TROP-2-positive OC by minimal incorporation of DFO to SG with less than one chelator per SG. Previous studies have also explored SG for imaging applications [12]; however, higher degrees of labeling have likely compromised the ADC’s pharmacokinetics as seen by high liver uptake. Prior work has shown liver accumulation when the degree of labeling is dramatically increased [14]. Meanwhile, other studies have focused on developing novel imaging agents for detecting TROP-2 [27, 29] without the burden of drug conjugates. By PET/CT and terminal biodistribution studies this work has demonstrated that [^89^Zr]Zr-DFO-SG effectively targets TROP-2-positive tumors in subcutaneous and intraperitoneal OVCAR3 models, with classical antibody uptake kinetics between 24 h and 144 h post-injection (Fig. 1D-E). Overall, the tumor retention with [^89^Zr]Zr-DFO-SG, emphasizes the potential of SG and potentially other TROP-2 antibodies as a radiopharmaceutical.

Among the radiopharmaceutical conjugates explored here [^177^Lu]Lu-DTPA-SG could be administered as a theranostic ADC option, but repeat dosing was needed and overall survival was similar to standard of care SG in this model (Fig. 2D). Since the beta emitter was not curative, the transition to ^225^Ac with a higher LET became necessary. Two conjugation strategies for the macropa chelator, click and direct, were explored for superiority as a ^225^Ac ADC with SG. All of the immunoconjugates were found to bind to recombinant human TROP-2, showing no difference in immunoreactivity (Supplemental Fig. 4) except a slightly lower uptake with [^177^Lu]Lu-DTPA-SG. [^225^Ac]Ac-mcp-Direct-SG intriguingly had the highest non-specific uptake while [^225^Ac]Ac-mcp-Click-SG was one of the lowest. The biodistribution (Fig. 3A) and therapeutic (Fig. 3B) outcomes of both radioimmunoconjugates show clearly that the direct TFP conjugation of SG targeted the tumor best and demonstrated major tumor reduction with prolonged survival.

Importantly, the success of the direct method is a combination of the minimal conjugation of SG, 1.6 vs. 8.3 moieties attached, on top of the reported 7.6 drug conjugates already, and different linkages used with PEG_4_ vs. PEG_7_-Tz-TCO. Hypothetically if SG was modified with only one TCO-Tz, tumor targeting would have improved. In a recent orthogonal study, another TROP-2 full length antibody was conjugated with 7.8 TCOs and found that more than one DOTA-PEG-Tz chelator per antibody could reduce tumor targeting but did not change BLI binding affinity (**Pratt**,** CCR 2026 Accepted**). Together these data highlight the combination of chelator, charge, number of overall modifications (including drug conjugates and unused TCO moieties), are key considerations in ADC theranostic design. In particular, the TFP and NHS conjugation methods are preferrable due to amide bond formation over a thiocyanate conjugation which yields a labile thioether bond. Thus TFP and NHS linkages limit radionuclide cleavage from the antibody, though daughter release is a separate issue. Also, the TFP method requires a longer reaction time compared to thiocyanate conjugation potentially allowing finer control of the degree of conjugation. Here minimal modification maintains tumor targeting with the ADC SG and limits liver uptake, affirming earlier work with an unconjugated antibody showing the overall limitations to an antibody degree of labeling [14].

Limitations of this study include the use of a slow growing OVCAR3 model, limiting the chance to create follow up studies such as a full biodistribution of [^177^Lu]Lu-DTPA-SG as well as a repeat survival study comparing a [^225^Ac]Ac-Click-SG with one to three TCO-Tz clicks per antibody. This study used 8.3 TCOs on average coupled with ~ 3.3 TCO-Tz clicks per SG, a 0.4:1 molar ratio of Tz: TCO. Another limitation of this study was that [^225^Ac]Ac-mcp-Direct-SG received two therapeutic doses in the study duration, while other arms such as [^225^Ac]Ac-mcp-Click-SG received only one. The decision for a second dose relied on the stable CBC results and not on study symmetry. Future studies into fractionation and cycles of therapy are warranted. Another limitation in this study was the slow tumor growth meaning therapy arms had to be staged as mice were available. Still, therapy arm averages were within the specified 100–350 mm^3^ size range at the start of each therapy cohort. Also, SG which is known to release SN-38 molecules once in solution, will create some variation with the number of molecules present on each SG for the conjugates produced. Future work could readily be done with more stable ADCs such as datopotamab-deruxtecan. In addition, the implementation of TCO for click chemistry also requires a two-step reaction, first to attach the TCO and then with a pre-defined stoichiometry, add the Tz moiety. Furthermore, the TCO-Tz click should be conducted within a few days as the isomerization of axial transcyclooctene inactivates the click reaction with Tz. Furthermore, in each imaging and therapy arm, variation in uptake and as such therapeutic response was also variable especially in the [^177^Lu]Lu-DTPA-SG group. Given the degree of IHC heterogeneity seen in Fig. 1B, OVCAR3 responses highlight the complexity of tumor heterogeneity, resistance, and individual tolerance to therapy [30].

Overall, this study highlights the importance of selecting an appropriate conjugation method and degree of labeling to maximize an ADC’s efficacy and safety by tuning the degree of conjugation and limiting off target delivery of either the radionuclide or drug conjugate. Given the regression and tolerability with [^225^Ac]Ac-mcp-Direct-SG, direct chelator conjugation offers a promising approach for future ADC targeted alpha therapies. In particular, the use of the TFP conjugation is a one-step reaction unlike the prior conjugation of TCO and the required secondary click reaction in a low enough ratio to not overburden SG with TCO and or the clicked macropa. While this study was conducted in only one ovarian cancer model, SG is approved for use in metastatic breast cancer highlighting other potential indications for this work. In addition TROP-2 has been identified as high in 54.8% of high grade serous ovarian cancer samples, with 57.7% as well for endometroid cancers [31] meaning additional populations could take advantage of a [^225^Ac]Ac-mcp-Direct-SG therapy, even where SG therapy alone may have been toxicity limiting [32].

## Conclusions

New imaging and therapeutic agents are crucial for the detection and elimination of high-grade serous OCs, given the silent progression until later stages. By minimally modifying the FDA-approved SG with various relevant chelator systems, SG has been transformed into a viable PET imaging agent as well as a radiopharmaceutical therapy agent with [^177^Lu]Lu-DTPA-SG or [^225^Ac]Ac-mcp-Direct-SG for TROP-2 positive OCs. Using the TFP linker chemistry, the conjugation of SG becomes straightforward, with conjugation and radiolabeling with Actinium-225 done at 37 °C with high radiochemical conversion. By limiting the bioconjugation of SG to nearly one chelate, radiopharmaceutical therapy could be attained with SG without sending the conjugate to the liver and spleen. Here by minimally modifying SG, a new imaging and successful alpha therapy stratagem is presented for OC.

## Supplementary Information

Below is the link to the electronic supplementary material.

Supplementary Material 1

## Data Availability

All data within this manuscript and supplement is available upon request to the corresponding author. Materials related to this manuscript, such as pretargeted chelates can be available upon reasonable request to Jason S. Lewis via email at Lewisj2@mskcc.org.

## References

[1] Siegel RL, et al. Cancer statistics, 2025. CA Cancer J Clin. 2025;75(1):10–45. 39817679 10.3322/caac.21871 PMC11745215

[2] Feeney L, et al. Liquid biopsy in ovarian cancer: catching the silent killer before it strikes. World J Clin Oncol. 2020;11(11):868–89. 33312883 10.5306/wjco.v11.i11.868 PMC7701910

[3] Momenimovahed Z, et al. Ovarian cancer in the world: epidemiology and risk factors. Int J Womens Health. 2019;11:287–99. 31118829 10.2147/IJWH.S197604 PMC6500433

[4] Meric-Bernstam F, et al. Efficacy and safety of trastuzumab Deruxtecan in patients with HER2-Expressing solid tumors: primary results from the DESTINY-PanTumor02 phase II trial. J Clin Oncol. 2024;42(1):47–58. 37870536 10.1200/JCO.23.02005 PMC10730032

[5] Bogani G, et al. Mirvetuximab soravtansine-gynx: first antibody/antigen-drug conjugate (ADC) in advanced or recurrent ovarian cancer. Int J Gynecol Cancer. 2024;34(4):469–77. 38101816 10.1136/ijgc-2023-004924

[6] Moore KN, et al. Phase III, randomized trial of Mirvetuximab Soravtansine versus chemotherapy in patients with platinum-resistant ovarian cancer: primary analysis of FORWARD I. Ann Oncol. 2021;32(6):757–65. 33667670 10.1016/j.annonc.2021.02.017

[7] Nelson BE, Meric-Bernstam F. Leveraging TROP2 Antibody-Drug conjugates in solid tumors. Annu Rev Med. 2024;75:31–48. 37758237 10.1146/annurev-med-071322-065903

[8] Goldenberg DM, Stein R, Sharkey RM. The emergence of trophoblast cell-surface antigen 2 (TROP-2) as a novel cancer target. Oncotarget. 2018;9. 10.18632/oncotarget.25615 PMC6034748 29989029

[9] Qiu S, et al. Targeting Trop-2 in cancer: recent research progress and clinical application. Biochim Biophys Acta Rev Cancer. 2023;1878(4):188902. 37121444 10.1016/j.bbcan.2023.188902

[10] Lopez S, et al. Preclinical activity of sacituzumab Govitecan (IMMU-132) in uterine and ovarian carcinosarcomas. Oncotarget. 2020;11:5. 10.18632/oncotarget.27342 PMC7007291 32082489

[11] Fenn KM, Kalinsky K. Sacituzumab govitecan: antibody-drug conjugate in triple-negative breast cancer and other solid tumors. Drugs Today. 2019;55(9):575-585. 10.1358/dot.2019.55.9.3039669 PMC7303962 31584574

[12] Huang W et al. Preclinical evaluation of zirconium-89 labeled anti-Trop2 antibody-drug conjugate (Trodelvy) for imaging in gastric cancer and triple-negative breast cancer. Eur J Nucl Med Mol Imaging. 2025;52(7):2369-2383. 10.1007/s00259-025-07106-4 PMC12119234 39878898

[13] Huang W et al. ImmunoPET imaging of Trop2 expression in triple-negative breast cancer using [(64)Cu]Cu-NOTA-Trodelvy-F(ab’)(2). Eur J Nucl Med Mol Imaging. 2025;52(9):3223-3237. 10.1007/s00259-025-07167-5 PMC12221780 39994021

[14] Sharma SK, et al. A systematic evaluation of antibody modification and (89)Zr-Radiolabeling for optimized Immuno-PET. Bioconjug Chem. 2021;32(7):1177–91. 32197571 10.1021/acs.bioconjchem.0c00087 PMC9423892

[15] Nejadmoghaddam MR, et al. Antibody-Drug conjugates: possibilities and challenges. Avicenna J Med Biotechnol. 2019;11(1):3–23. 30800238 PMC6359697

[16] Mack KN et al. Interrogating the theranostic capacity of a MUC16-Targeted antibody for ovarian cancer. J Nucl Med. 2024;65(4):580-585. 10.2967/jnumed.123.266524 PMC10995531 38485271

[17] Mack KN et al. Pretargeted alpha therapy in MUC16-positive high-grade serous ovarian cancer. Nucl Med Biol. 2025;140–1:108976. 10.1016/j.nucmedbio.2024.108976 PMC12404150 39615062

[18] Herrero Alvarez N, et al. Recent advances in radiometals for combined imaging and therapy in cancer. ChemMedChem. 2021;16(19):2909–41. 33792195 10.1002/cmdc.202100135

[19] Wei Q, et al. The promise and challenges of combination therapies with antibody-drug conjugates in solid tumors. J Hematol Oncol. 2024;17(1):1. 38178200 10.1186/s13045-023-01509-2 PMC10768262

[20] Bauer D et al. Examination of the PET in vivo generator (134)Ce as a theranostic match for (225)Ac. Eur J Nucl Med Mol Imaging. 2024;51:4015–4025. 10.1007/s00259-024-06811-w PMC12121724 38940841

[21] Marcu L, Bezak E, Allen BJ. Global comparison of targeted alpha vs targeted beta therapy for cancer: in vitro, in vivo and clinical trials. Crit Rev Oncol/Hematol. 2018;123:7–20. 29482781 10.1016/j.critrevonc.2018.01.001

[22] Bauer D, et al. Click chemistry: a transformative technology in nuclear medicine. Nat Protoc. 2023;18(6):1659–68. 37100960 10.1038/s41596-023-00825-8 PMC10293801

[23] Thiele NA, et al. An Eighteen-Membered macrocyclic ligand for Actinium-225 targeted alpha therapy. Angew Chem Int Ed Engl. 2017;56(46):14712–7. 28963750 10.1002/anie.201709532

[24] Jelovac D, Armstrong DK. Recent progress in the diagnosis and treatment of ovarian cancer. CA Cancer J Clin. 2011;61(3):183–203. 21521830 10.3322/caac.20113 PMC3576854

[25] Lheureux S, Braunstein M, Oza AM. Epithelial ovarian cancer: evolution of management in the era of precision medicine. CA Cancer J Clin. 2019;69(4):280–304. 31099893 10.3322/caac.21559

[26] Huang W et al. Immuno-PET/CT imaging of Trop2 with [(18)F]AlF-RESCA-T4 differentiates lung cancer from inflammation. J Nucl Med. 2024;65(12):1904-1910. 10.2967/jnumed.124.268751 39542697

[27] Pratt EC et al. Pretargeted Trop-2 ImmunoPET for rapid, selective detection of pancreatic tumors. Clin Cancer Res. 2025;31(13):2719–2726. 10.1158/1078-0432.CCR-24-3098 PMC12213220 39841860

[28] Flynn MJ, Ledermann JA. Ovarian cancer recurrence: is the definition of platinum resistance modified by PARPi and other intervening treatments? The evolving landscape in the management of platinum-resistant ovarian cancer. Cancer Drug Resist. 2022;5(2):424–35. 35800366 10.20517/cdr.2022.13 PMC9255242

[29] Huang W, et al. ImmunoPET imaging of Trop2 expression in solid tumors with nanobody tracers. Eur J Nucl Med Mol Imaging. 2024;51(2):380–94. 37792026 10.1007/s00259-023-06454-3

[30] Koltai T, Fliegel L. The relationship between Trop-2, chemotherapeutic Drugs, and chemoresistance. Int J Mol Sci. 2023;25(1):87. 10.3390/ijms25010087 PMC10779383 38203255

[31] Porter JM, et al. Antibody drug conjugate targets are highly differentially expressed across the major types of ovarian cancer. Eur J Cancer. 2025;224:115522. 40446758 10.1016/j.ejca.2025.115522

[32] Bardia A et al. Final results from the randomized phase III ASCENT clinical trial in metastatic Triple-Negative breast cancer and association of outcomes by human epidermal growth factor receptor 2 and trophoblast cell surface antigen 2 expression. J Clin Oncol. 2024;42(15):1738-1744. 10.1200/JCO.23.01409 PMC11107894 38422473

