# Supplementary material for: Minimal chemical modification enables alpha/beta radiolabeling of sacituzumab govitecan for targeted therapy in high grade serous ovarian cancer
Source: Eur J Nucl Med Mol Imaging. 2026 Feb 28;53(7):4587–96. doi: 10.1007/s00259-026-07820-7 (PMC13197279; doi:10.1007/s00259-026-07820-7)
Supplement: Supplementary file 1 — Supplementary Material 1 [file 259_2026_7820_MOESM1_ESM.docx]

**Title: Minimal Chemical Modification Enables Alpha/Beta Radiolabeling of Sacituzumab Govitecan for Targeted Therapy in High Grade Serous Ovarian Cancer**

**Short Title:** Radiopharmaceutical Therapy with Sacituzumab-Govitecan

**Authors:** Angelique Loor^1±^, Kyeara N. Mack^1,2±^, David Bauer^1^, Aidan Ingham^1^, Edwin C. Pratt^1*#^ and Jason S. Lewis ^1,2,3,*^

**Affiliations:**

^1^ Molecular Pharmacology Program, Memorial Sloan Kettering Cancer Center, New York, New York.

^2^ Department of Pharmacology, Weill Cornell Graduate School of Medical Sciences, Weill Cornell Medicine, New York, New York.

^3^ Department of Radiology, Memorial Sloan Kettering Cancer Center, New York, New York.

^±^Authors contributed equally

*Corresponding Authors

^#^New Affiliation is Stony Brook University Cancer Center, Stony Brook, New York.

**Financial Support:** This work was supported by the following grants: National Institutes of Health R01 CA215700 and R01 EB033000 (to JG), R35 CA232130 (to JSL), S10 OD016207 (to Pat Zanzonico, MSKCC), and P30 CA08748 (to Selwyn Vickers MSKCC). ECP has been supported by the National Institutes of Health F32 CA268912 and K99 CA276804.

**Corresponding Authors:** ECP and JSL can be reached at [edwin.pratt@stonybrookmedicine.edu](mailto:edwin.pratt@stonybrookmedicine.edu) and [lewisj2@mskcc.org](mailto:lewisj2@mskcc.org). Authors can be reached by mail at 1275 York Avenue, ZRC 2031, New York, NY, 10065

**Key words: TROP-2; PET Imaging, Radioimmunotherapy, Ovarian Cancer, Targeted Alpha Therapy**

**Supplemental Data:**

**Ovarian Cancer Cell lines**

Human ovarian adenocarcinoma OVCAR3 was purchased from the American Type Culture Collection (ATCC). All cell lines were authenticated by short tandem repeat (STR) profiling at Memorial Sloan Kettering (MSK) and were checked twice a year for mycoplasma. The cultures were maintained under sterile conditions at 37 °C with 5% CO₂ in a humidified environment. OVCAR3 cells were cultured in RPMI medium supplemented with 20% fetal calf serum (FCS), 10 mM HEPES, 2 mM l-glutamine, 1 mM sodium pyruvate, 1.5 g/L sodium bicarbonate, 4.5 g/L glucose, 0.01 mg/mL bovine insulin, 100 units/mL penicillin, and 100 µg/mL streptomycin.

**Serum stability**

[^89^Zr]Zr-DFO-SG was prepared as mentioned in the methods radiolabeling section. The radioimmunoconjugate was then diluted into human serum 10:1 and incubated on a thermomixer at 37 °C for 24, 48, 72, and 144 h. [^89^Zr]Zr-DFO-SG conversion was determined by SG iTLC plates and EDTA (50 mM, pH 5.5) mobile phase.

**Dosimetry**

The dosimetry analysis was performed using the OLINDA/EXM. Each arm was computed using a trapezoidal fitting model provided to the biodistribution time points, with an alpha weighting of 5 and a total dose projected for 37 kBq of ^225^Ac administered. As TROP-2 is rapidly internalized, the fitting model assumed no clearance from the tumor.

**Intraperitoneal Implantation**

For intraperitoneal implantation, 5 million OVCAR3 cells in 150 µL 1:1 medium/BD Matrigel were implanted into the abdomen. Imaging of IP-implanted mice was conducted at 2-, 3-, 4-, and 5-months post-implantation, allowing nearly 10 half-lives for ^89^Zr to decay. After 5 months, the mice were euthanized, and tissue was preserved in O.C.T. compound for sectioning.

**Immunohistochemistry**

Tumor tissue samples were harvested and fixed in 10% neutral buffered formalin. Fixed tissues were embedded in paraffin blocks, sectioned and then stained using the Ventana system with a mouse and human reactive TROP-2 antibody (Abcam Catalog ab214488) at 0.5 µg/mL and an anti-rabbit secondary.

**Bead binding assay**

HisPur Ni-NTA beads were conjugated with human TROP-2 as described previously[1] and based on [2]. [^89^Zr]Zr-DFO-SG (3.7 kBq) was added to the suspension and mixed for 1 h at RT. Blocking was done 30 minutes prior with 100-fold unlabeled SG. Cells were washed in triplicate and collected by centrifugation at 500 g for 5 minutes before gamma counting. Uptake was compared to 100% of the activity administered.

**Cell Binding assay**

One million OVCAR3 cells were collected and counted before resuspension into PBS in an Eppendorf tube. [^89^Zr]Zr-DFO-SG (3.7 kBq) was added to the suspension and mixed for 1 h at RT. Blocking was done 30 minutes prior with 100-fold unlabeled SG. Cells were washed in triplicate and collected by centrifugation at 500 g for 5 minutes before gamma counting. Uptake was compared to 100% of the activity administered.

**Autoradiography IP model**

Tumors and livers were collected at the time of sacrifice and preserved frozen in O.C.T. and sectioned for autoradiography. Slides were exposed at -20°C for a week before exposure with a Typhon-3000 slide scanner at a 50 µm scan resolution.

**Statistical Analysis**

Data were analyzed using GraphPad Prism v10. Unpaired, two-tailed *t* tests assessed bead-binding; one-way ANOVA with Tukey-Kramer correction was used for organ uptake in biodistribution experiments. Survival was analyzed via a log rank (Mantel–Cox) test. *P* < 0.05 was considered significant.

**Pathology Analysis**

**[^177^Lu]Lu-DTPA-SG cohort**

Two of the seven mice treated with [^177^Lu]Lu-DTPA-SG developed subcutaneous carcinomas with variable intratumoral necrosis. Similarly, three of the seven mice treated with SG also had subcutaneous carcinomas. In contrast, there was no macro or microscopic evidence of tumor xenograft in mouse 2 and mouse 5.

Ovarian cortical atrophy and follicular degeneration, observed in this study, were considered nonspecific off-target radiotoxic effects on germ cells. These lesions have been previously documented in other radioimmunotherapy studies involving ^177^Lu and other radionuclides. Additionally, the ovarian mass noted in mouse 1 was diagnosed as a cystadenocarcinoma, based on its histomorphological appearance and the arrangement of neoplastic epithelial cells. Such ovarian tumors have been sporadically reported in different inbred mouse strains, with incidence appearing to increase with age. Spontaneous cystadenocarcinomas are known to spread to peritoneal organs via ovarian invasion of adjacent tissues. Given the off-target radiation effects observed in the ovarian cortex, it cannot be ruled out that the treatment may have induced neoplastic transformation of epithelial cells in the ovary.

Bone marrow from all mice showed adequate presence of myeloid and erythroid precursors, along with megakaryocytes. While differences in myeloid-to-erythroid (M:E) ratios may exist between animals, such variations could not be determined through histopathological evaluation.

Hematology: Red blood cell (RBC) parameters indicated no anemia in the submitted samples. Platelet counts were within normal limits for mice 2 through 7. However, mouse 1 exhibited a marked reduction in platelet and total white blood cell counts. Additionally, the spleen in mouse 1 showed an increased number of erythroid precursors, correlating with reticulocytosis noted in peripheral blood.

Serum Biochemistry: Mild increases in aspartate aminotransferase (AST) levels were observed in mice 1, 4, and 5. While elevated AST levels can indicate significant inflammation or damage to the liver, cardiac, or skeletal muscle, this study did not observe such damage. Previous studies reported mild AST elevations due to the intracardiac blood collection method. Slightly elevated globulin levels were noted in mice 1, 2, 3, 4, and 6, potentially due to antigenic stimulation of lymphocytes and plasma cells from the antibody treatments. Mice 7 is within normal range for AST and globulin levels.

**[²²⁵Ac]Ac-click-SG and [²²⁵Ac]Ac-direct-SG cohorts**

Female nude mice in the click cohort were administered 1 μCi of [²²⁵Ac]Ac-click-SG, while mice in the direct cohort received an initial dose of 1 μCi, followed by 0.7 μCi of [²²⁵Ac]Ac-direct-SG. Mice were grouped by conjugation chemistry: three mice in the TCO-Tz (click chemistry) linker group, while eight mice were in the direct TFP linker group. All other components of the formulation remained consistent across groups.

Histological evaluation of kidney tissue revealed dose-dependent radiotoxicity characterized by moderate to severe tubulointerstitial lesions in all treatment groups. These findings are consistent with known actinium-225–associated nephrotoxicity. Given that TROP-2 is expressed in murine renal tubular epithelium during development, potential on-target binding of the TROP-2-directed antibody may contribute to renal localization and injury. Notably, mice in the click group showed more pronounced histopathological damage. Serum biochemistry revealed moderate to marked elevation in BUN in one of three mice in this group, while creatinine remained within normal limits across all animals, suggesting early-stage azotemia in a subset of treated mice. Elevated BUN was also observed in mice from the direct group, though to a lesser extent.

Liver sections for the click cohort of mice showed moderate to marked hepatocellular degeneration and hypertrophy, indicating a hepatotoxic response. These alterations are potentially related to the linker or altered pharmacokinetics. In contrast, liver tissue from mice in the direct cohort was unremarkable, with no evidence of degeneration or hypertrophy. Serum liver enzyme analysis supported these findings: two of three mice from the click cohort exhibited elevated ALT, AST, and ALP levels, which is indicative of hepatocellular injury. Additionally, elevations in AST, along with increases in ALT and ALP, are indicative of hepatic injury, with ALT and ALP elevations being particularly consistent with liver-specific damage. The exclusive appearance of these histological and biochemical liver changes in the click cohort strongly indicates the linker chemistry as a contributing factor.

Ovarian lesions consistent with radiotoxicity to germinal cells were observed across all groups, reflecting the known off-target effects of alpha-particle emitters. Histology of the OVCAR3 tumors confirmed carcinoma with variable necrosis and inflammation, with higher tumor burden observed in click cohort (3/3) compared to the direct cohort (4/8). No evidence of metastasis was detected in any group. Lymphoid hyperplasia in the spleen and lymph nodes, and myeloid hyperplasia in the bone marrow, were observed across groups and likely represent reactive changes to treatment.

One mouse in the direct group developed lymphoma three months post-administration and was euthanized for pathology. The submandibular lymph nodes were enlarged (left: 0.6 × 0.4 × 0.5 cm; right: 0.7 × 0.8 × 0.3 cm). There were two pale tan and firm subcutaneous tumors in the right shoulder area (1^st^ tumor: 1.0 × 0.4 × 0.4 cm and 2^nd^ tumor 0.6 × 0.4 x 0.3 cm). No other gross abnormalities were noted. The subcutaneous nodules in the right flank and right and left shoulders were consistent with lymphoma. This lymphoma was disseminated in multiple nodes, causing lymphadenomegaly and infiltrating multiple tissues, including the bone marrow. Neoplastic lymphocytes were small to medium compared to murine red blood cells. Based on the histomorphology and distribution of the neoplastic cell population, the lymphoma was likely T-cell in origin. IHC for T and B cell markers is recommended to determine the nature of the neoplastic cell population. Tubular lesions in the kidneys were secondary to radiotoxicity from ^225^Ac.

**Synthesis of mcp-PEG_4_-TFP (direct)**

All chemicals not further specified were purchased from Sigma-Aldrich and used as received. As mentioned in the Methods section, p-NCS-Bn-DFO and p-NCS-CHX-A"-DTPA were purchased by Macrocyclics, while Mcp-PEG_8_-Tz had been previously synthesized from our group (*Eur. J. Nucl.* *Med. Mol. Imaging.* **2024**, *51*, 4015). As for mcp-PEG_4_-TFP, synthesized compounds were characterized via ^1^H and ^13^C{^1^H} NMR spectroscopy, as well as MS-ESI. The final compound, macropa-triazole-PEG_4_-TFP, was additionally identified via HR-MS and ^19^F NMR spectroscopy.

HPLC purifications were performed on a Shimadzu UFLC HPLC system equipped with a DGU-20A_3_ degasser, an SPD-M20A UV detector, an LC-20AB pump system, and a CBM-20A communication BUS module using a C18 reversed phase XTerra® Preparative MS OBD column (10 μm, 19×250 mm) at a constant flowrate of 8 mL/min (solvent A: 0.1% trifluoroacetic acid (TFA) in H_2_O, solvent B: 0.1% TFA in MeCN). A solvent gradient of 10→70% (solvent B) in 25 minutes was used.

Except for the final reaction, the strategy used to synthesize mcp-PEG_4_-TFP (Supplemental Scheme 1) largely follows the synthetic route previously established for macropa-propargyl (**6**) (*Cancers*. **2021**, *13*, 1974). Within this study, compound **6** was resynthesized with some minor variations, such as different purification steps and a switch to bromo-based compound **3** (opposed to the chloro-based analogue).

Briefly, the synthesis of mcp-PEG_4_-TFP can be described as follows. First, 3-bromoprop-1-yne is added onto dimethyl 4-hydroxypyridine-2,6-dicarboxylate via an S_N_2 reaction to form **1**. Compound **1** then undergoes an asymmetric reduction using NaBH_4_ to form **2**. The hydroxyl group of **2** is then brominated with PBr_3_ to produce the electrophilic picolinate arm, **3**, which is subsequently added to the secondary amine of **4** via a S_N_2 reaction, forming **5**. In the penultimate reaction, LiOH is added to **5** to convert the methyl ester groups into carboxylates, thereby forming **6**. Finally, a copper-catalyzed, alkyne-azide click reaction is carried out in DMSO to form the desired compound: macropa-triazole-PEG_4_-TFP or ‘mcp-PEG_4_-TFP’. A more detailed experimental write up of these synthetic reactions is also provided below.


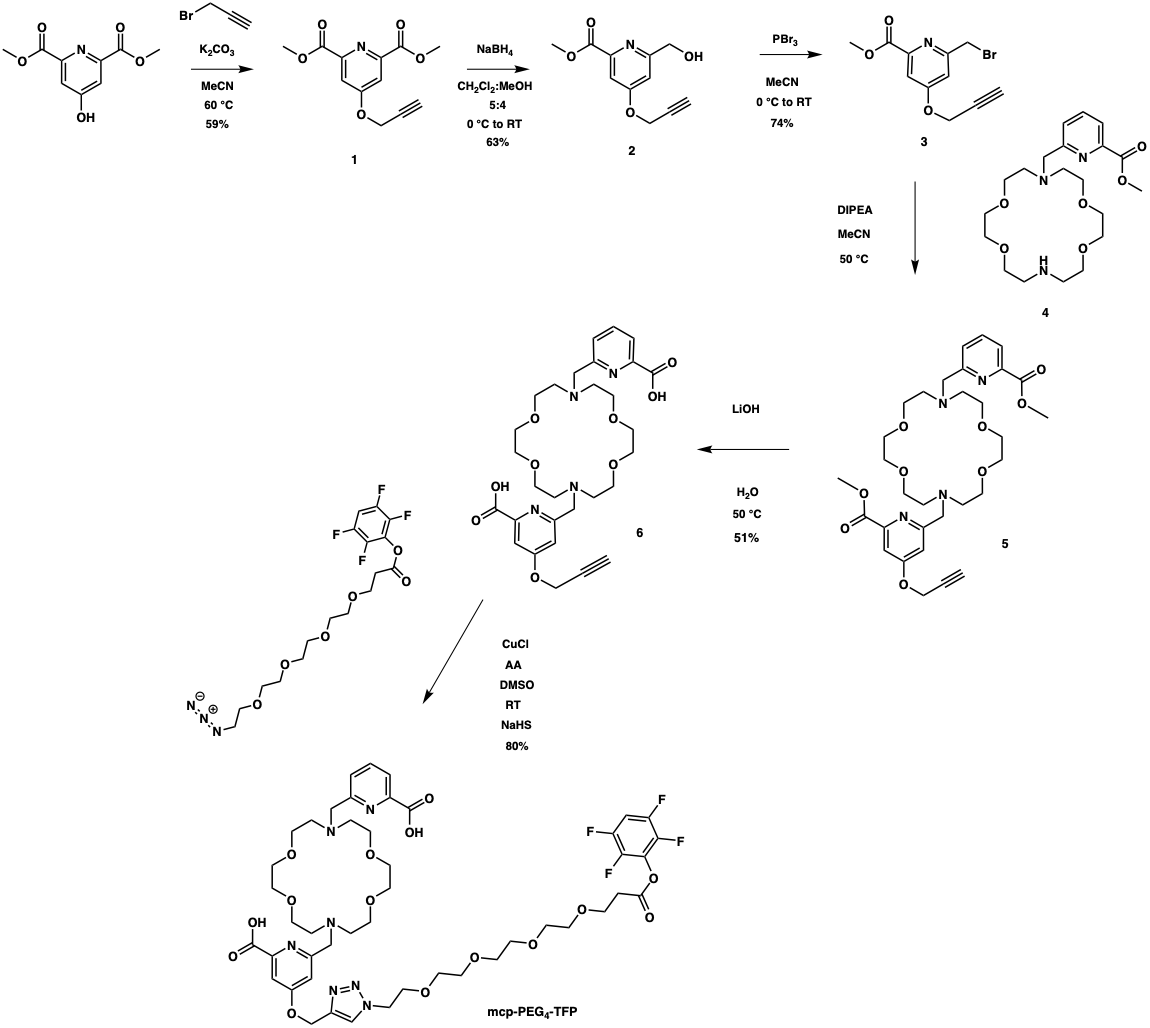


**Supplemental Scheme 1.** Synthesis of macropa-triazole-PEG_4_-TFP or ‘mcp-PEG_4_-TFP’

*Dimethyl 4-(prop-2-yn-1-yloxy)pyridine-2,6-dicarboxylate (****1****).* Dimethyl 4-hydroxypyridine-2,6-dicarboxylate monohydrate (0.650 g, 3.08 mmol) was dissolved in MeCN (15 mL). As the reaction mixture was heated up to 60 ℃, K_2_CO_3_ (1.4 g, 10.1 mmol, 3.3 equiv) and propargyl bromide (1 mL, 9 mmol, 3 equiv) were added. After 24 h, TLC showed the reaction had gone to completion. As a result, the reaction mixture was cooled to room temperature, filtered, and had its volatiles removed in vacuo. The remaining crude mixture was redissolved in CH_2_Cl_2_ (15 mL) and washed with H_2_O (15 mL). The aqueous layer was then further extracted with CH_2_Cl_2_ (2 x 20 mL). The organic layers were combined, dried with Na_2_SO_4_, filtered, and dried in vacuo to give **1** as a pure white solid product (0.416 g, 1.67 mmol, 59%). LR-MS: calcd for [C_12_H_11_NO_5_ + H]^+^, 250.1; found [M + H]^+^, 250.1. The NMR spectra were also in accordance to previously published: *Cancers*, 2021, **13**, 1974.

*Methyl 6-(hydroxymethyl)-4-(prop-2-yn-1-yloxy)picolinate (****2****).* Compound **1** (1.9 g, 7.6 mmol) was dissolved in CH_2_Cl_2_ and MeOH (50 mL : 50 mL), and the solution was cooled to 0 ℃. NaBH_4_ (0.3 g, 7.9 mmol, 1 equiv) was added in portions over one hour while the reaction mixture was in an ice bath. The ice bath was then removed, and the reaction was monitored by TLC. After one more hour, the reaction mixture was diluted with CH_2_Cl_2_ (50 mL) and quenched using saturated NH_4_Cl (30 mL). The organic layer was collected, and the aqueous layer was further extracted with CH_2_Cl_2_ (3 x 60 mL). The organic layers were combined, dried with MgSO_4_, filtered, and concentrated in vacuo to give a yellow oil. The oil was redissolved in CH_2_Cl_2_, loaded onto silica, and purified by column chromatography (*CombiFlash* R_f_ automated column system; 40 g HP silica column; A: CH_2_Cl_2_, B: MeOH; 0% B to 5% B). The fractions containing pure product were collected and concentrated to afford **2** as a white solid (1.07 g, 4.8 mmol, 63%). LR-MS: calcd for [C_11_H_11_NO_4_ + H]^+^, 222.1; found [M + H]^+^, 222.0. The NMR spectra were also in accordance to previously published: *Cancers*, 2021, **13**, 1974.

*Methyl 6-(bromomethyl)-4-(prop-2-yn-1-yloxy)picolinate (****3****).* Compound **2** (1.05 g, 4.75 mmol) was dissolved in MeCN (40 mL) and the solution was cooled to 0 ℃. PBr_3_ (0.54 mL, 5.7 mmol, 1.2 equiv) was then added over 5 minutes, and the colorless solution became yellow. After two hours, the reaction mixture was quenched with saturated Na_2_CO_3_ solution (40 mL) over a few minutes. EtOAc (60 mL) was added, and the organic layer was collected. EtOAc (2 x 60 mL) was then used to extract more product from the aqueous layer. The organic layers were combined, dried with MgSO_4_, filtered, and concentrated in vacuo to give **3** as a white solid (1.0 g, 3.5 mmol, 74%). ^1^H NMR (500 MHz, 298 K, CDCl_3_): *d* 7.67 (d, ^4^J = 2.5 Hz, 1H), 7.26 (d, ^4^J = 2.6 Hz, 1H), 4.82 (d, ^4^J = 2.5 Hz, 2H), 4.60 (s, 2H), 4.01 (s, 3H), 2.61 (t, ^4^J = 2.5 Hz, 1H). ^13^C{^1^H} NMR (125 MHz, 298 K, CDCl_3_): *d* 165.3, 165.3, 159.0, 149.4, 113.6, 111.6, 77.4, 76.6, 56.2, 53.3, 33.3. LR-MS: calcd for [C_11_H_10_BrNO_3_ + H]^+^, 284.0; found [M + H]^+^, 284.1.

*Mcp-propargly(****6****).*Methyl6-((1,4,10,13-tetraoxa-7,16-diazacyclooctadecan-7-yl)methyl)picolinate ×2 TFA (**4**) (110 mg, 0.17 mmol) was dissolved in MeCN (10 mL), and methyl 6-(bromomethyl)-4-(prop-2-yn-1-yloxy)picolinate (**3**) (60 mg, 0.2 mmol, 1.2 equiv) and DIPEA (0.1 mL, 0.6 mmol, 3 equiv) were added. The reaction mixture was heated to 50 ℃ and was left to stir overnight. The following day, the solvent was taken off in vacuo, leaving a crude yellow oil of **5**, which was then resuspended in H_2_O (2 mL). LiOH (100 mg, 4.2 mmol, 25 equiv) was added and the reaction mixture was heated to 50 ℃, stirring for one hour. The reaction was cooled to room temperature, and the pH adjusted to 3–5 with 10% TFA. The mixture was directly injected into the HPLC (200 µL per run). The collected fractions were combined, frozen, and lyophilized to obtain **6** as a white powder (70 mg, 0.086 mmol, 51%). ^1^H NMR (500 MHz, 298 K, CDCl_3_): *d* 8.10 (m, 1H), 8.09 (m, 1H), 7.76 (m, 1H), 7.67 (d, 1H), 7.39 (d, 1H), 4.97 (s, 2H), 4.79 (s, 2H), 4.73 (s, 2H), 3.94 (t, 8H), 3.68 (s, 8H), 3.65 (t, 8H), 3.07 (t, 1H). ^13^C{^1^H} NMR (125 MHz, 298 K, CDCl_3_): *d* 167.1, 166.4, 166.4, 151.1, 149.7, 148.5, 146.5, 140.1, 128.0, 125.3, 114.4, 112.3, 78.0, 76.8, 69.8, 63.9, 57.3, 57.2, 56.7, 54.6, 54.6. LR-MS: calcd for [C_29_H_38_N_4_O_9_ + H]^+^, 587.3; found [M + H]^+^, 587.5.

*Mcp-PEG4-TFP.* Compound **6** (5.93 mg, 8.14 µmol), N_3_-PEG_4_-TFP (Biopharma PEG, 4.30 mg, 9.77 µmol, 1.2 equiv.), Cu(I)Cl (1.21 mg, 12.2 µmol, 1.5 equiv.) and ascorbic acid (2.15 mg, 12.2 µmol, 1.5 equiv.) were dissolved in anhydrous DMSO (0.5 mL) using a 2.0 mL microcentrifuge tube as reaction vessel; the color changed from green to blue within 5 minutes. The mixture was reacted overnight at room temperature. The next day, NaHS (6.84 mg, 0.122 mmol, 15 equiv.) dissolved in 0.4 mL H_2_O was added to precipitate all copper ions; the reaction mixture turned black instantly. Without delay, the microcentrifuge tube was centrifuged at 12,000 × g for 3 minutes, and the supernatant was injected into the HPLC system (0.9 mL total volume). The collected fraction was frozen and lyophilized to obtain mcp-PEG_4_-TFP ×2 TFA as a white powder. (8.16 mg, 6.51 µmol, 80%). ^1^H NMR (500 MHz, 298 K, DMSO-d6): *d* 8.26 (s, 1H), 8.12 (t, ^3^J = 7.6 Hz, 1H), 8.09 (d, ^3^J = 7.7 Hz, 1H), 7.93 (m, 1H), 7.79 (d, ^3^J = 7.4 Hz, 1H), 7.74 (d, ^4^J = 2.4 Hz, 1H), 7.49 (d, ^4^J = 2.4 Hz, 1H), 5.37 (s, 2H), 4.68 (s, 2H), 4.60 (s, 2H), 4.55 (t, ^3^J = 5.2 Hz, 2H), 3.83 (s, 8H), 3.82 (t, ^3^J = 5.4 Hz, 2H), 3.75 (t, ^3^J = 5.9 Hz, 2H), 3.51 (m, 28H), 3.01 (t, ^3^J = 6.3 Hz, 2H). ^13^C{^1^H} NMR (125 MHz, 298 K, DMSO-d6): *d* 167.9, 166.1, 165.5, 165.3, 152.5, 151.1, 149.6, 147.7, 146.5, 144.6, 141.2, 139.2, 128.6, 127.8, 125.5, 124.5, 114.2, 111.1, 104.5, 69.8, 69.8, 69.7, 69.7, 69.6, 69.5, 69.4, 68.6, 65.6, 64.5, 61.8, 56.8, 53.4, 53.4, 49.5, 34.0. ^19^F NMR (213 MHz, 298 K, DMSO-d6): -139.3, -153.3.

**NMR spectra**


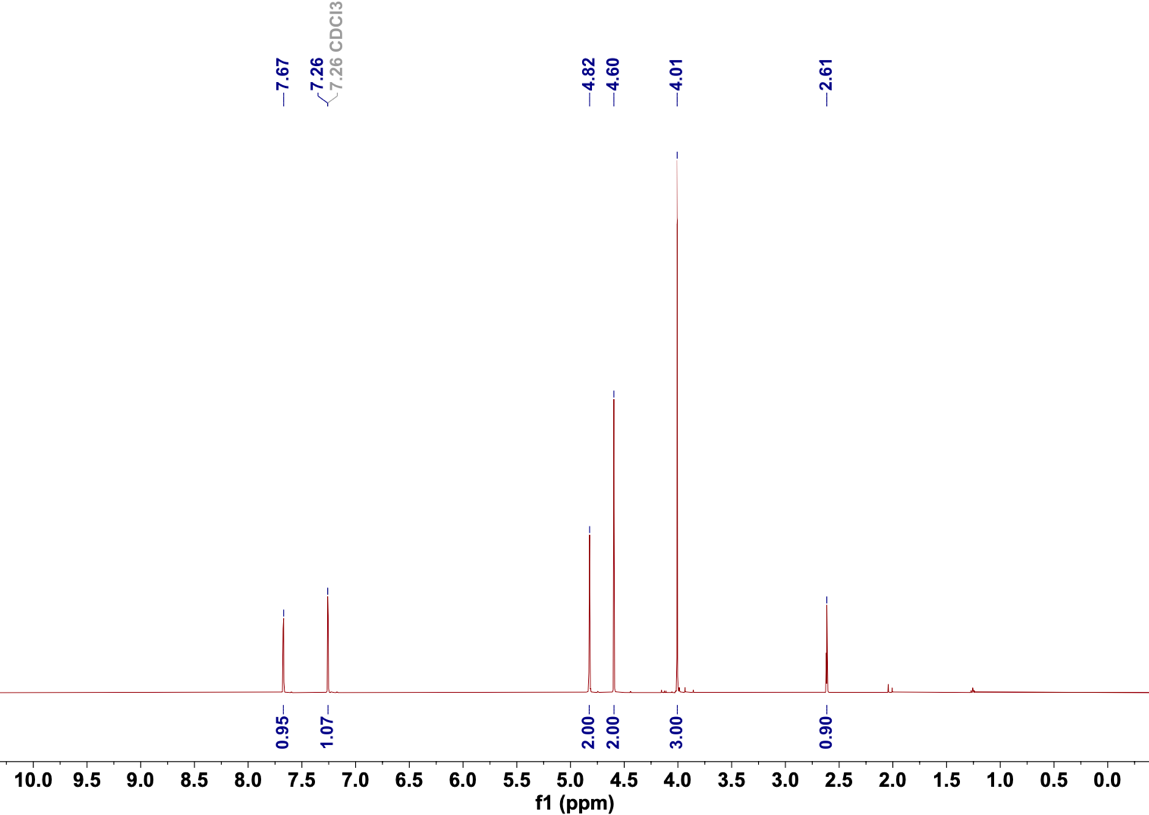


^1^H NMR spectrum of methyl 6-(hydroxymethyl)-4-(prop-2-yn-1-yloxy)picolinate **(3**) **(**500 MHz, CDCl_3_).


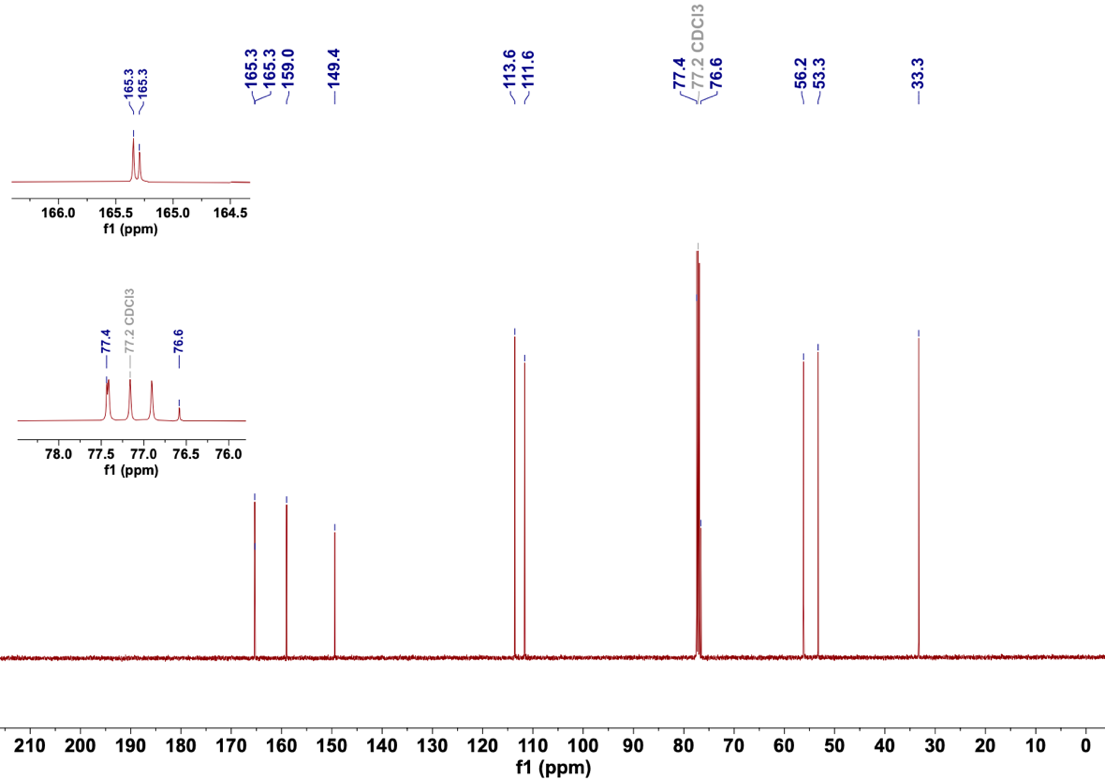


^13^C{^1^H} NMR spectrum of methyl 6-(hydroxymethyl)-4-(prop-2-yn-1-yloxy)picolinate **(3**) **(**125 MHz, CDCl_3_).


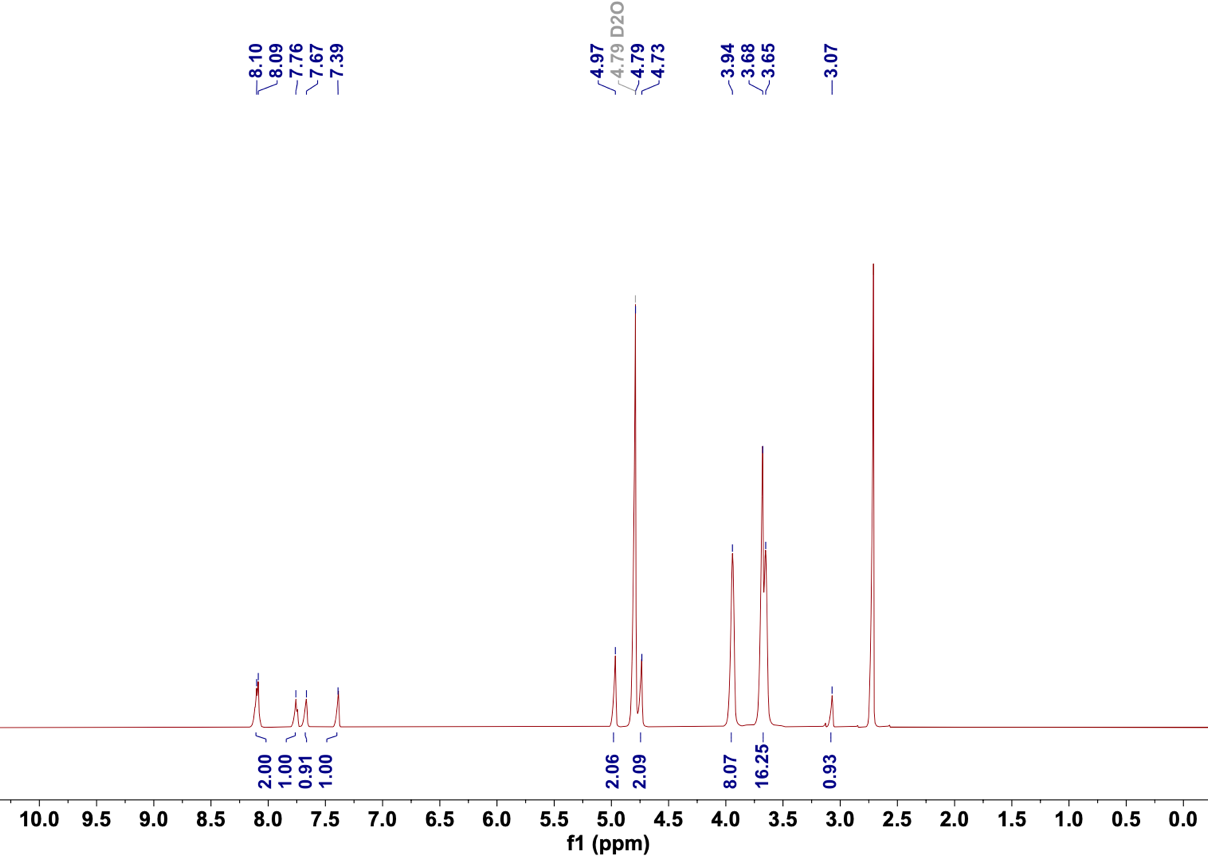


^1^H NMR spectrum of macropa-propargly-TFA salt (**6**) (500 MHz, D_2_O).


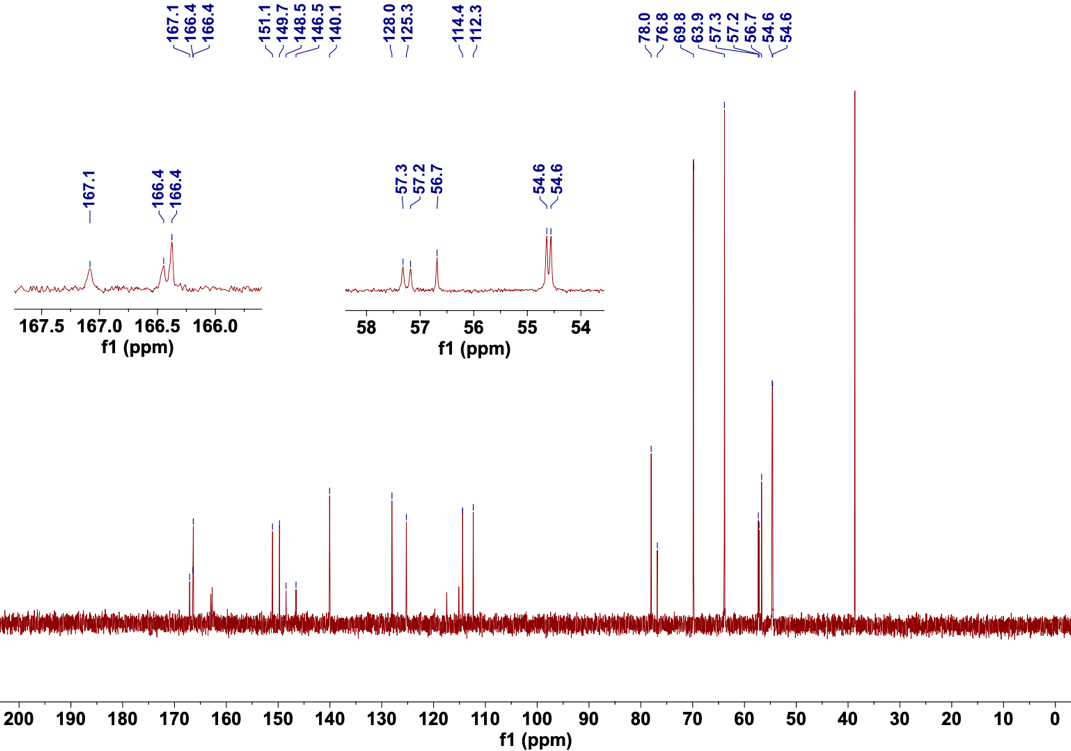


^13^C{^1^H} NMR spectrum of macropa-propargly-TFA salt (**6**) (125 MHz, D_2_O).


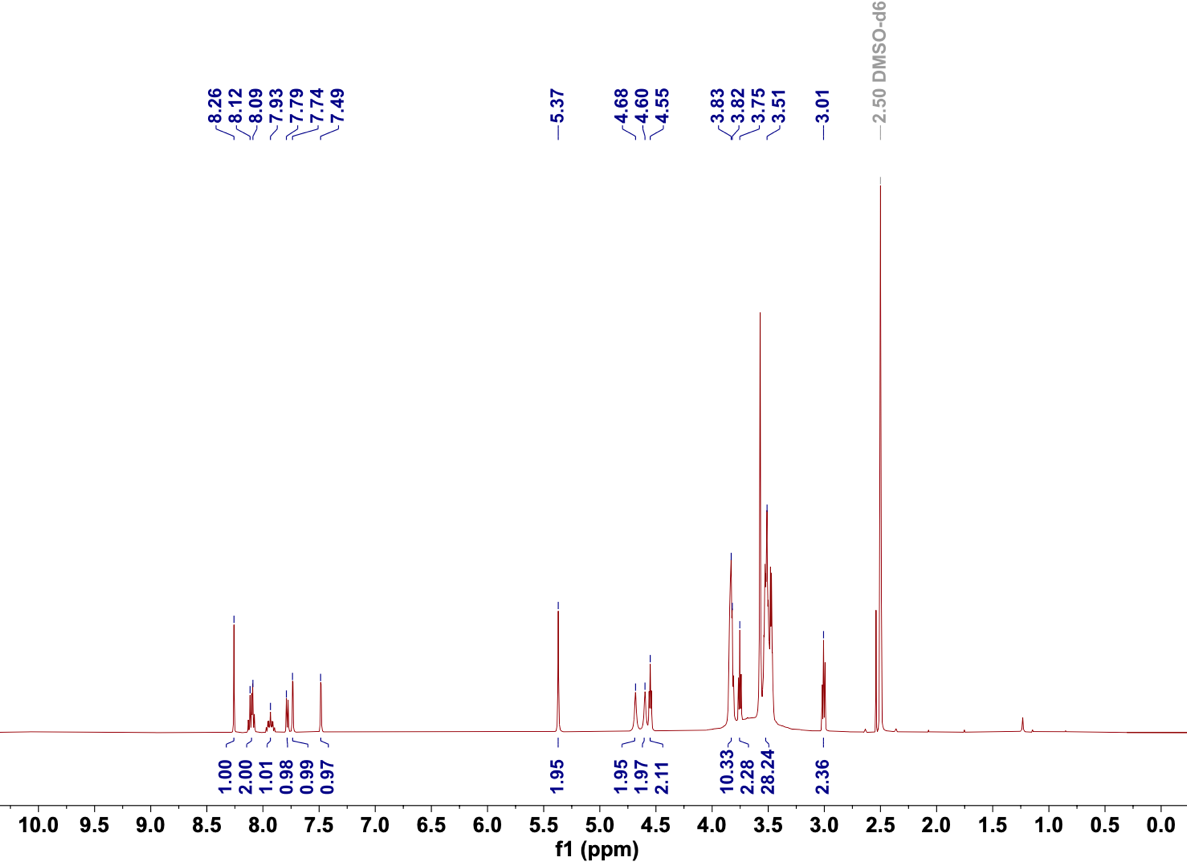


^1^H NMR spectrum of mcp-PEG_4_-TFP-TFA salt (500 MHz, DMSO-d6).


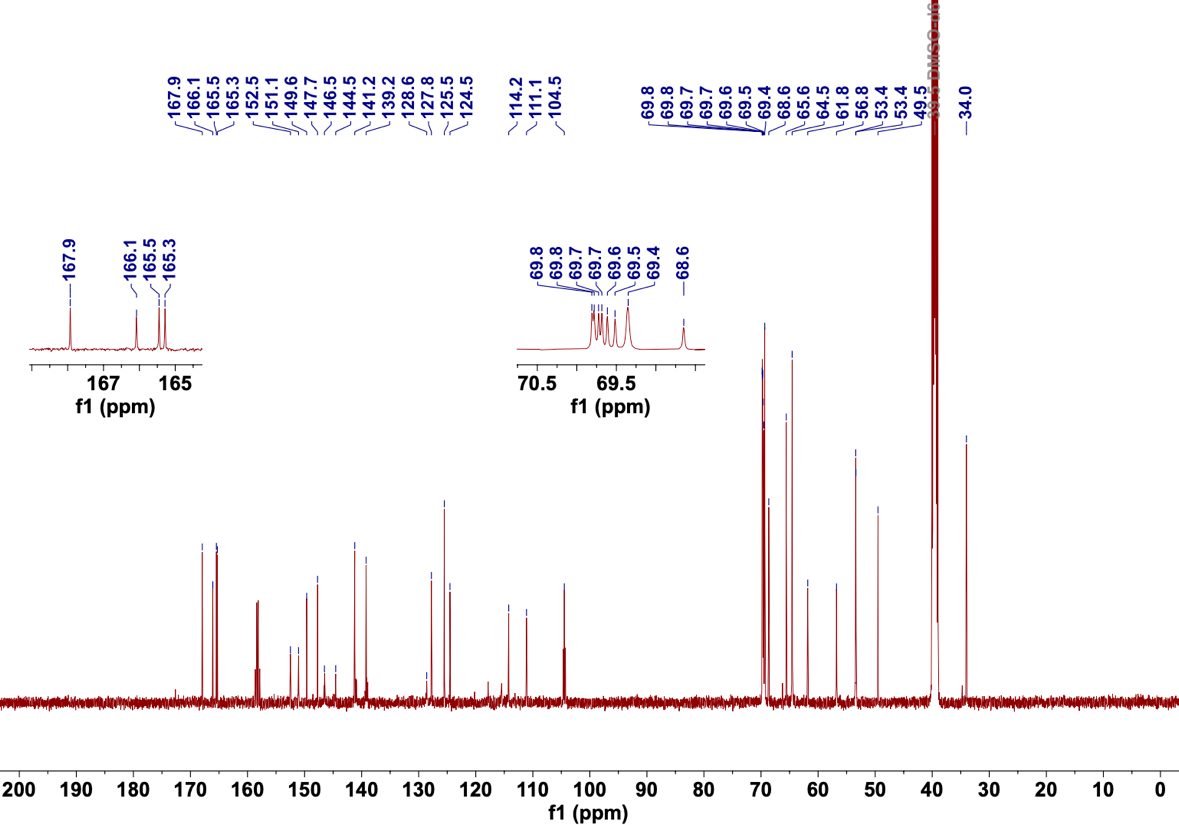


^13^C{^1^H} NMR spectrum of mcp-PEG_4_-TFP-TFA salt (125 MHz, DMSO-d6).


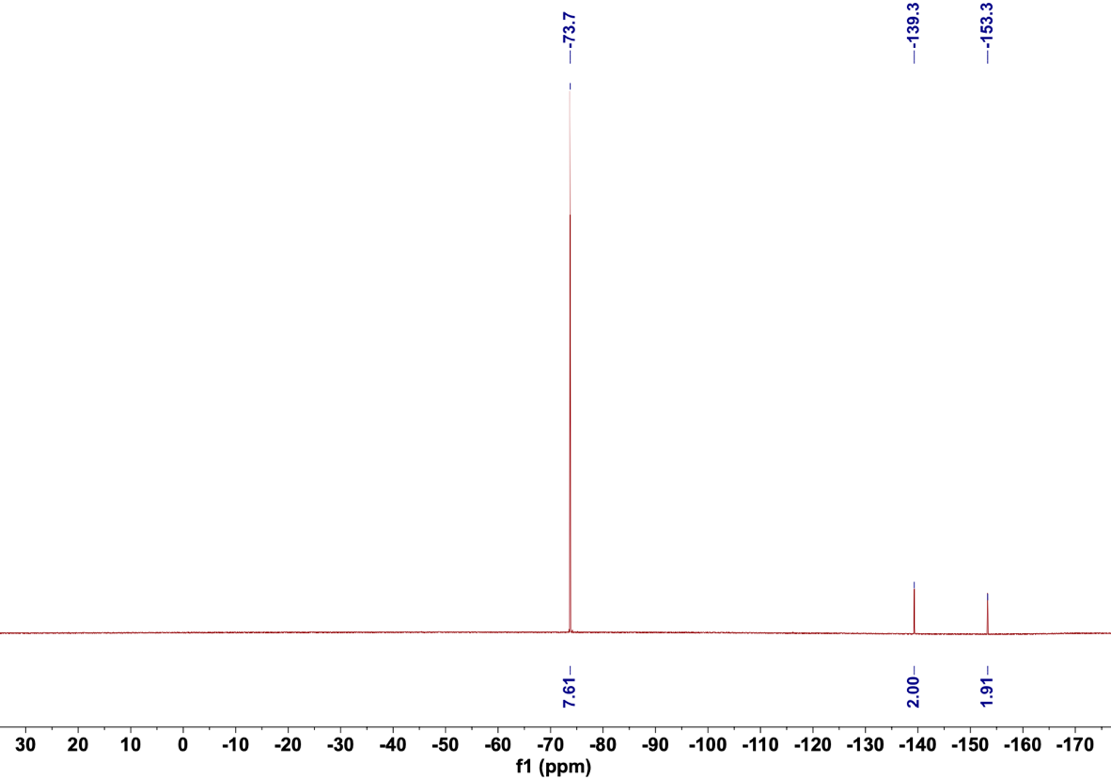


^19^F NMR spectrum of mcp-PEG_4_-TFP-TFA salt (213 MHz, DMSO-d6).


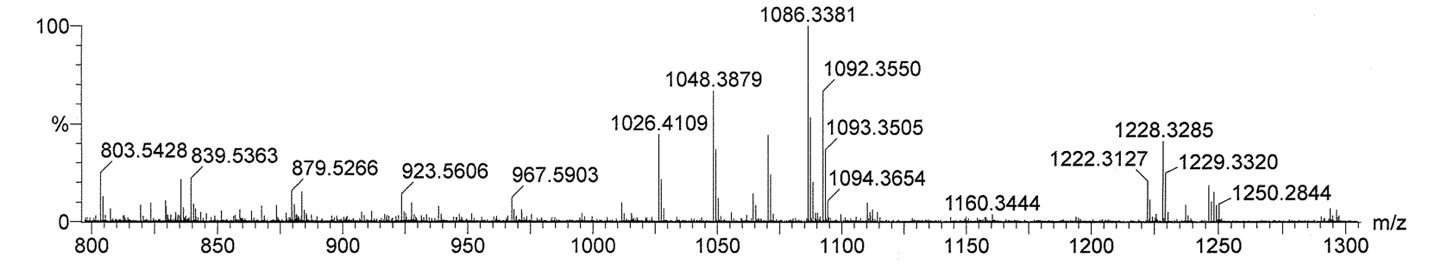


HRMS-ES^+^ of mcp-PEG_4_-TFP: calcd for [C_46_H_59_F_4_N_7_O_15_ + H]^+^, 1026.4084; found [M + H]^+^, 1026.4109

**Supplemental Figures:**


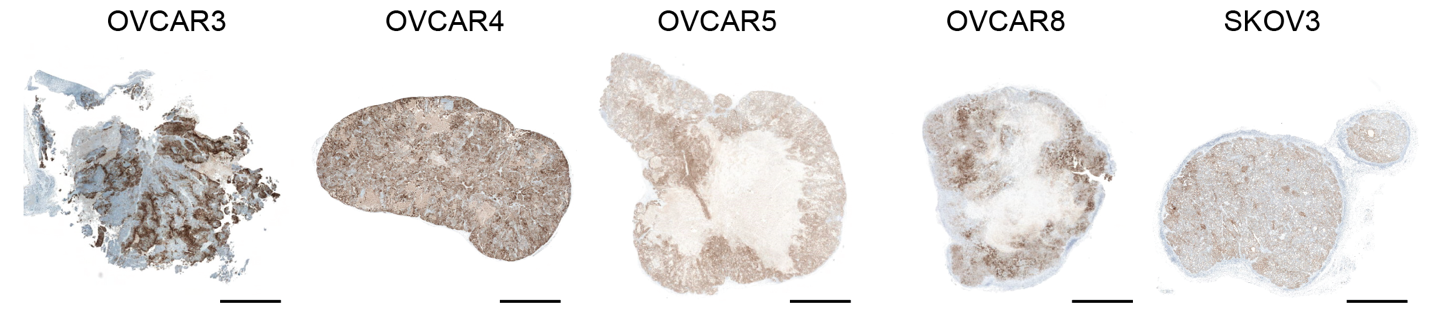
**Supplemental Figure 1. Immunohistochemistry (IHC)** presented in this figure demonstrated the levels of TROP-2 across five ovarian cancer cells (OVCAR3, OVCAR4, OVCAR5, OVCAR8, and SKOV3). OVCAR3 and OVCAR4 exhibit the strongest staining, indicating high TROP-2 expression, while OVCAR5, OVCAR8, and SKOV3 show moderate to low staining. Bar = 2 mm.

**Supplemental Figure 2: MALDI- TOF spectra of unmodified and modified SG. A)** Radio-instant thin layer chromatography shows high radiochemical conversion > 98% for [^225^Ac]Ac-mcp-direct-SG, [^225^Ac]Ac-mcp-click-SG, [^177^Lu]Lu-DTPA-SG, and [^89^Zr]Zr-DFO-SG. **B)** [^89^Zr]Zr-DFO-SG shows > 95% stability in human serum over 7 days at 37 °C. **C)** A fresh sample of unmodified SG shows a peak at ~ 109191 m/z, **D)** while an older batch of SG shows peak at ~ 108439 m/z, which calculates to an average release of ~2.6 SG. **E)** SG-DFO peak at ~104236.1 m/z, which calculates to an average of ~0.5 DFO molecules per antibody. **F)** TCO-SG (click) peak at ~79848 m/z, which calculates to an average of ~8.3 molecules per antibody. **G**) TFP-SG (direct) peak at ~80146 m/z, which calculates to an average of ~1.6 molecules per antibody.

**
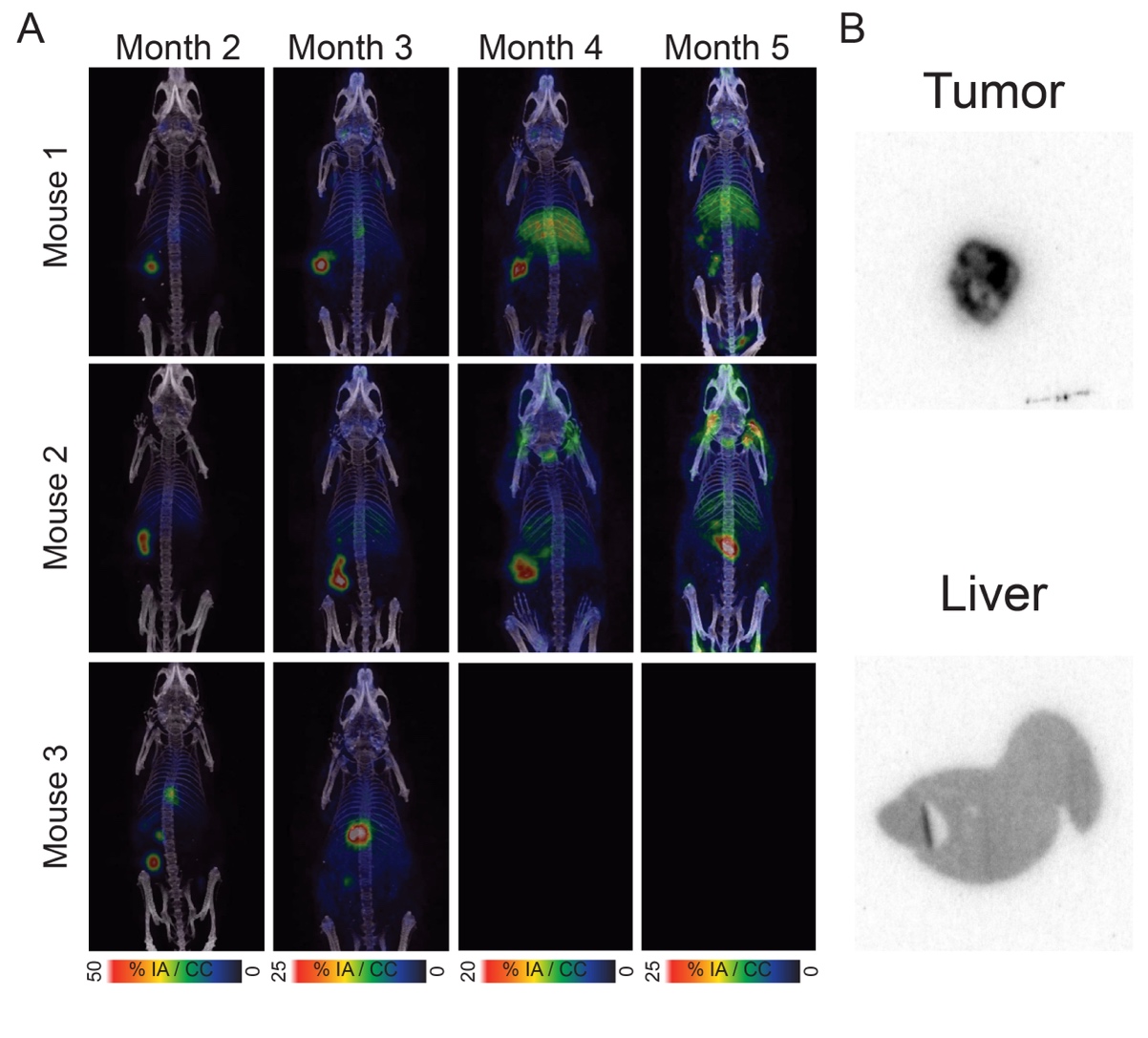
**

**Supplemental Figure 3. [^89^Zr]Zr-DFO-SG in an intraperitoneal model. A**) PET/CT imaging of OVCAR3 intraperitoneally implanted shows lesions with high uptake of the radiotracer through month 5. **B**) Autoradiography of mouse 1 showing uptake in the excised tumor while lower nonspecific uptake in the liver.

**Supplemental Figure 4. Bead binding assay to evaluate the antigen binding of radiolabeled SG using** four different radiolabeled SG conjugates— [⁸⁹Zr]Zr-DFO-SG (blue), [¹⁷⁷Lu]Lu-DTPA-SG (red), [²²⁵Ac]Ac-click-SG (green), and [²²⁵Ac]Ac-direct-SG (purple). The radiolabeled conjugates showed effective binding to target indicating that antigen specificity was preserved; additionally conjugates were blocked by a 100-fold excess of unlabeled SG yielding uptake similar to bead only conditions. Bar = mean, error = SEM, n=4 replicates per condition. P-values for graph * < 0.05, ** < 0.01, *** < 0.001, **** < 0.0001.


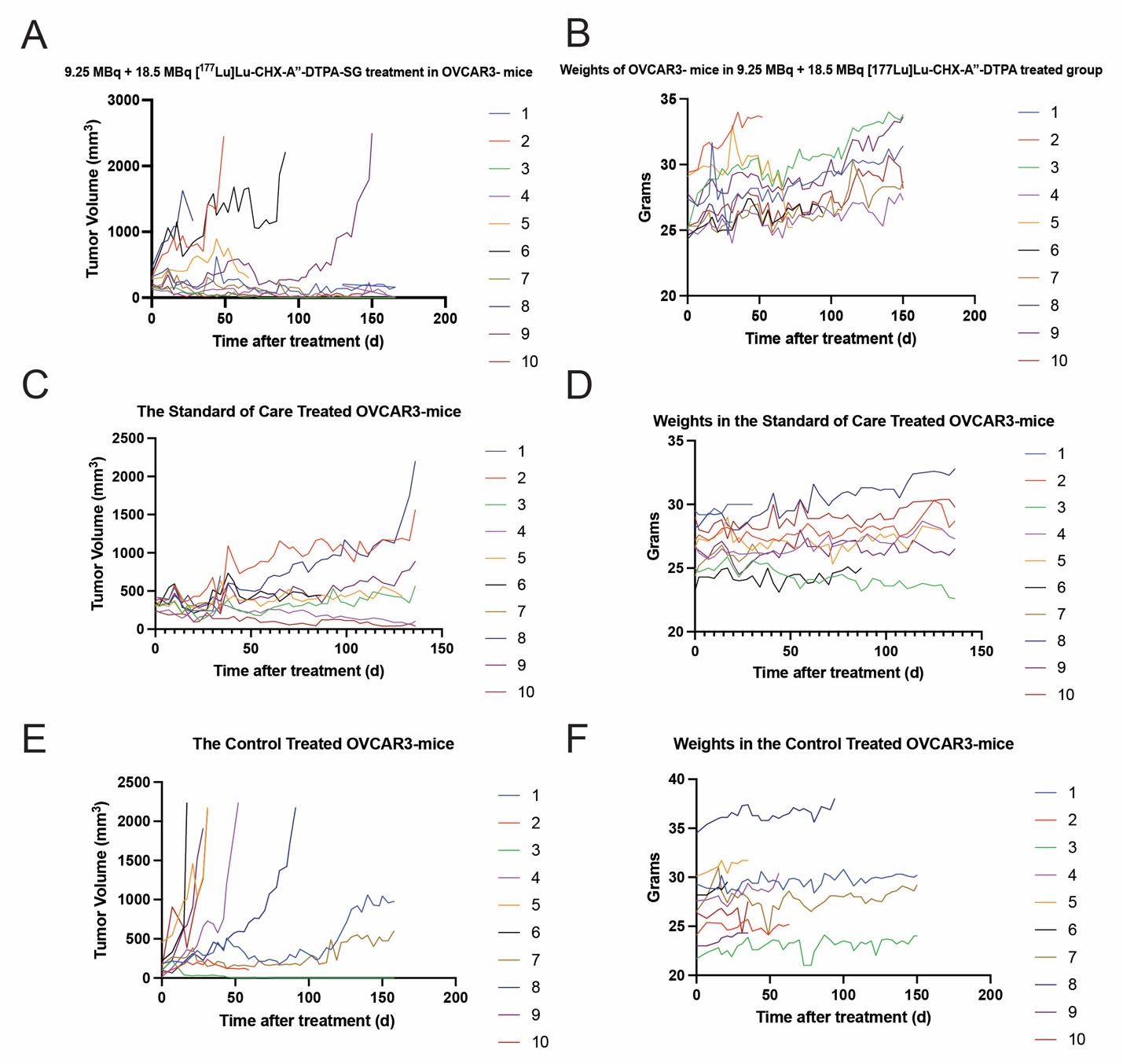


**Supplemental Figure 5. OVCAR3 mice in 9.25 MBq + 18.5 MBq [^177^Lu]Lu-DTPA-SG cohort shows A)** individual tumor volumes exhibit variability such as tumor regression over time, while others display regrowth. **B)** The mouse weights show no drastic weight loss > 20%, only slight fluctuation, and generally remaining between 25 and 35 grams.

**Supplemental Figure 6. Full ex-vivo terminal Biodistribution for** [^225^Ac]Ac-mcp-direct-SG cohort and [^225^Ac]Ac-mcp-click-SG cohort. Two-way ANOVA analysis on 168 h terminal biodistributions shows superior tumor uptake for the [^225^Ac]Ac-mcp-direct-SG cohort. The dosimetry of click and direct conjugations is based on terminal biodistribution values reported in Supplemental Table 1.

|  | Direct-TFP (Sv/µCi) | Click-TCO  (Sv/µCi) | Direct-TFP  % Relative Dose Deposited to Tumor | Click-TCO  % Relative Dose Deposited to Tumor |
| --- | --- | --- | --- | --- |
| Blood/Marrow | 9.65 | 0.85 | 17% | 28% |
| Heart | 2.38 | 0.80 | 4% | 27% |
| Lungs | 5.02 | 1.09 | 9% | 36% |
| Liver | 9.82 | 19.34 | 18% | 644% |
| Spleen | 3.55 | 7.25 | 6% | 241% |
| Pancreas | 1.11 | 0.36 | 2% | 12% |
| Small Intestine | 1.0 | 0.61 | 2% | 20% |
| Colon | 0.76 | 0.57 | 1% | 19% |
| Kidneys | 3.77 | 2.19 | 7% | 73% |
| Muscle | 0.81 | 0.18 | 1% | 6% |
| Cortical Bone | 1.52 | 1.48 | 3% | 49% |
| Tumor | 55.35 | 3.00 | 100% | 100% |

**Supplemental Table 1.** Dosimetry estimates for 1.0 µCi of ^225^Ac using SG's direct and click conjugates were calculated based on terminal biodistribution values from Supplemental Figure 6. These estimates used a trapezoidal fitting model and an alpha particle weighting of 5, with results reported as total dose deposited (Sv) for 37 kBq (1.0µCi) of ^225^Ac.

Supplemental References:

1. Pratt, E.C., et al., *Pretargeted Trop-2 immunoPET for rapid, selective detection of pancreatic tumors.* Clinical Cancer Research, 2025.

2. Sharma, S.K., et al., *A rapid bead-based radioligand binding assay for the determination of target-binding fraction and quality control of radiopharmaceuticals.* Nucl Med Biol, 2019. **71**: p. 32–38.
